# Differences in Ex-Gaussian Parameters from Response Time Distributions Between Individuals with and Without Attention Deficit/Hyperactivity Disorder: A Meta-analysis

**DOI:** 10.1007/s11065-023-09587-2

**Published:** 2023-03-06

**Authors:** Marcos Bella-Fernández, Marina Martin-Moratinos, Chao Li, Ping Wang, Hilario Blasco-Fontecilla

**Affiliations:** 1 Hospital Universitario Puerta de Hierro Majadahonda, Madrid, Spain; 2 Universidad Autónoma de Madrid, Madrid, Spain; 3 Universidad Pontificia de Comillas, Madrid, Spain; 4 CIBERSAM Madrid, Madrid, Spain; 5 ITA Mental Health, Madrid, Spain

**Keywords:** ADHD, Reaction times, Ex-Gaussian, Inter-trial variability

## Abstract

Attention Deficit Hyperactivity Disorder (ADHD) is one of the most prevalent neurodevelopmental disorders in childhood and adolescence. Differences in reaction times (RT) in cognitive tasks have been consistently observed between ADHD and typical participants. Instead of estimating means and standard deviations, fitting non-symmetrical distributions like the ex-Gaussian, characterized by three parameters (*µ, σ*, and *τ*), account for the whole RT distributions. A meta-analysis is performed with all the available literature using ex-Gaussian distributions for comparisons between individuals with ADHD and controls. Results show that *τ* and *σ* are generally greater for ADHD samples, while *µ* tends to be larger for typical groups but only for younger ages. Differences in *τ* are also moderated by ADHD subtypes. *τ* and *σ* show, respectively, quadratic and linear relationships with inter-stimulus intervals from Continuous Performance Test and Go/No Go tasks. Furthermore, tasks and cognitive domains influence the three parameters. Interpretations of ex-Gaussian parameters and clinical implications of these findings are also discussed. Fitting ex-Gaussian distributions to RT data is a useful way to explore differences between individuals with ADHD and healthy controls.

**Supplementary Information:**

The online version contains supplementary material available at 10.1007/s11065-023-09587-2.

## Introduction

Attention Deficit Hyperactivity Disorder (ADHD) is one of the most prevalent neurodevelopmental disorders in childhood and adolescence, with a prevalence ranging from 4 to 8% (Polanczyk et al., 2014). ADHD is characterized by three core symptoms, namely, inattention, hyperactivity, and impulsivity (Barkley, 1997). Furthermore, patients with ADHD show impairments in several cognitive domains which impact daily activities. In the context of laboratory tasks, it has been consistently observed that individuals with ADHD show larger intra-individual variability in response times (RT) compared to healthy controls (Castellanos et al., 2006; Kofler et al., 2013; Levy et al., 2018; Tamm et al., 2012). This increased intra-individual variability has been proposed as a candidate endophenotype for ADHD (Castellanos et al., 2005; Henríquez-Henríquez et al., 2015; Karalunas et al., 2014; Lin et al., 2015; Salunkhe et al., 2020, 2021).

Means from RT distributions are often used in clinical research, but means alone do not account for the shape of the distribution (Dawson, 1988; Heathcote et al., 1991). Also, estimating means and standard deviations implicitly assumes that RT distributions are close to normal distributions, but empirical RT distributions seldom resemble normal curves. Rather, these distributions are usually positively skewed (Luce, 1986; Van Zandt, 2000). Using the mean or the median as central tendency statistics alone may conduce to biases and increase the risk of falsely rejecting null hypotheses (Morís Fernández & Vadillo, 2020; Rousselet & Wilcox, 2020). Furthermore, the mean and the standard deviation of RT distributions are strongly correlated (Luce, 1986; Wagenmakers & Brown, 2007). If the standard deviation, as a measure of intra-individual RT variability, is larger for people with ADHD, then this effect may also inflate the RT means for people with ADHD.

**Fig. 1:**
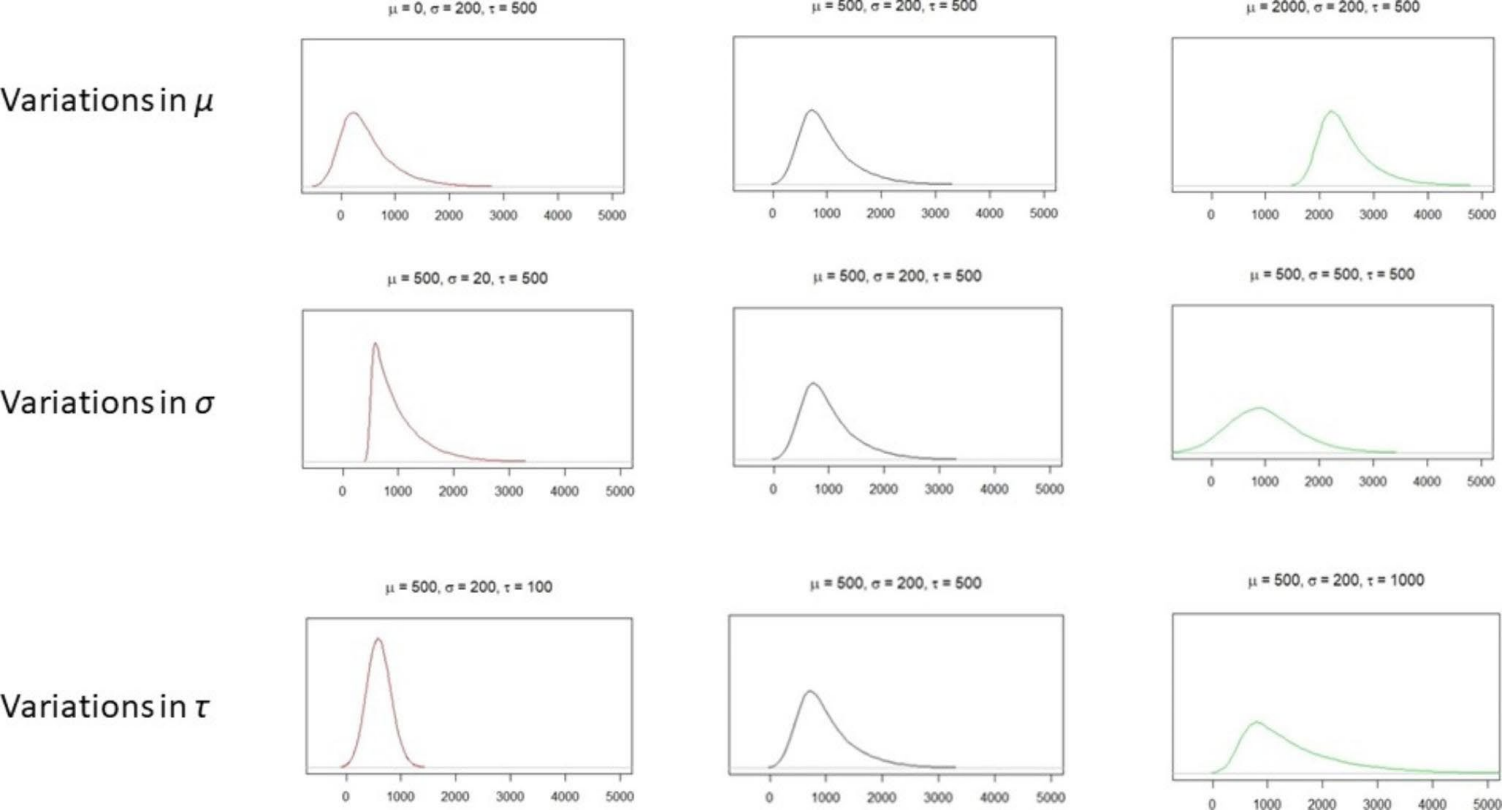
Shapes of ex-Gaussian distribution as parameter values are altered. The rows correspond to parameters *µ*, *σ*, and *τ*, respectively. From left to right, the parameter value goes larger. It can be seen that parameter *µ* mainly affects the position of the distribution. Parameter *σ* is mostly related to the width (dispersion) of the left part of the distribution, and greater values for parameter *σ* lead to wider distribution. Parameter *τ* affects the height of the left part of the distribution and the thickness of the tail, and larger values of *τ* lead to lower left parts and heavier tails. Graphs were made using “gamlss.dist” package for R (Stasinopoulos et al., 2021)

Among other alternative approaches (Ging-Jehli et al., 2021), using a theoretical distribution to describe and compare the shapes of different RT distributions has been proposed (Castellanos et al., 2006; Van Zandt, 2000). The most widely used theoretical distribution in ADHD research is the ex-Gaussian distribution. The ex-Gaussian distribution is the convolution of the Gaussian and the exponential distributions. It is defined by three parameters: *µ, σ*, and *τ*. Parameters *µ* and *σ* derive from the Gaussian mean and standard deviation and are related to faster, more regular responses. *τ* is related to atypically slow responses. Figure 1 describes in more detail the effect of the parameter values on the shape of the ex-Gaussian distribution. Unlike the mean and standard deviation, fitting ex-Gaussian distributions to empirical RTs allows RTs to be separated into a speed component, *µ*, and two variability components, *σ* and *τ* (Heathcote, 1996). These components are independent of each other, unlike RT mean and variance (Salunkhe et al., 2020). Furthermore, as discussed below, *σ* and *τ* have different meanings. Nonetheless, fitting ex-Gaussian models to RT data is not always recommended. First, a minimum of 100 observations is necessary to achieve reasonably reliable estimates (Heathcote, 1996; Lacouture & Cousineau, 2008; Van Zandt, 2000). Also, for participants who respond inconsistently, ex-Gaussian fit to RT data may be poor due to their proximity to normal distribution (Lacouture & Cousineau, 2008; Yamashita et al., 2021).

The seminal work by Leth-Steensen et al. (2000) first used the ex-Gaussian distribution in order to perform the “response time distributional approach”, which takes into account the whole RT distribution (and not merely mean and standard deviation) by comparing the ex-Gaussian parameter values for ADHD patients and healthy controls. They found that children with ADHD presented larger *τ* values than controls, which they named “tau effect”. This effect was attributed to a greater proportion of slow reactions from children with ADHD compared to controls. Since then, many studies applied the ex-Gaussian distribution to account for the whole RT distributions when comparing patients with neurodevelopmental disorders and control groups. Although the “tau effect” is generally confirmed (Karalunas et al., 2014; Kofler et al., 2013; Salunkhe et al., 2020), the effect of the two other parameters is less clear. Variables such as age (Galloway-Long et al., 2021; van Belle, van Hulst, et al., 2015; van Belle, van Raalten, et al., 2015), gender (Patros et al., 2018; Seymour et al., 2016) or cognitive task or domain (Epstein et al., 2011; Ging-Jehli et al., 2021) have been hypothesized to modulate these differences in RTs.

Although the ex-Gaussian distribution is often used as a purely descriptive tool for empirical reaction time distributions (Fitousi, 2020; Matzke & Wagenmakers, 2009; Rieger & Miller, 2020), investigating the correlates of ex-Gaussian parameters to cognitive processes is tempting, as this information would help to improve parameter interpretation and diagnosis through tasks using RT distributions. For instance, Yamashita et al. (2021) suggested that exGaussian distribution separated reaction times in a strategy factor (*µ*), a variability factor (*σ*), and a long-tail factor (*τ*).

In this sense, parameter *τ* has received the most attention, consistent with the greater relevance of *τ* in ADHD research. Several interpretations have been proposed. The most common interpretation of parameter *τ* is that longer *τ* reflects more frequent and pronounced attentional lapses during task performance (Leth-Steensen et al., 2000; Salunkhe et al., 2020). Other possible meanings for *τ* have been proposed. Based on state-regulation model (Sergeant, 2005), parameter *τ* may also be related to cognitive energy regulation impairments, which are hypothesized to be larger for people with ADHD. In constrast, studies based on information accumulation models suggest that the *τ* parameter may be related to impaired speed of information accumulation in patients with ADHD (Fitousi, 2020; Matzke & Wagenmakers, 2009). Research on the relationship between ex-Gaussian parameters and race models partly support the relationship between *τ* and information accumulation rates. Parameter *µ* has a more straightfoward interpretation. Variations in *µ* displace the RT distribution without altering its shape, thus suggesting that *µ* is related to reaction speed. In general terms, lower *µ* values reflect faster responses and higher values correspond to slower responses. *µ* may be influenced by speed-accuracy trade-offs (Yamashita et al., 2021). Lower values for *µ* have been related to impulsive behavior in ADHD patients. Last, parameter *σ* has been hypothesized to be related to motor timing or impaired response preparation (Leth-Steensen et al., 2000), perhaps as a consequence of poor neuro-modulation (Salunkhe et al., 2020; Yamashita et al., 2021) found a moderate correlation between *σ* and both omission and comission errors, which led these researchers to hypothesize a relationship between *σ* values and mind-wandering.

The aim of the present meta-analysis was to synthesize the available evidence on the ex-Gaussian distribution parameters estimated from RT data comparing between people with ADHD and controls, to examine the effect of ADHD on the whole RT distribution, and to examine the influence of potential moderator variables.

## Methods

### Search Strategy and Syntaxes

We conducted searches on databases PubMed, PsycInfo, PsycArticles, Embase, Cochrane, Web of Science, and Scopus. Search strings were similar to “ex-Gaussian” OR “exGaussian” (free language) AND “Attention Deficit/Hyperactivity Disorder”, including terms from the thesaurus when applicable. We also searched for grey literature in OpenGrey, OpenDOAR, WorldCat, and Google Scholar in order to reduce the risk of publication bias (Conn et al., 2003; McAuley et al., 2000; Siddaway et al., 2019). Searches were last performed on 7th April 2022. This meta-analysis was registered in PROSPERO (CRD42021277339).

### Inclusion and Exclusion Criteria

In the present meta-analysis, we included studies that (a) had observational or quasi-experimental designs with at least one sample of patients diagnosed with ADHD and one control group, (b) included estimations for at least one of the three ex-Gaussian parameters and contrast of differences between patients with ADHD and controls, and (c) provided sufficient information to extract or estimate Cohen’s d as effect size. Studies that did not provide contrasts between ADHD group and control group or did not provide sufficient data to estimate an effect size assimilable to Cohen’s d were excluded. Non-primary studies (e.g. reviews and meta-analyses) were also excluded. Lastly, for *σ* and *τ* estimations, only studies with at least 30 participants on each group were included (see “Data extraction” section below).

Two authors (MBF and MMM) independently selected studies through the Rayyan platform. Disagreements were resolved reaching a consensus.

### Data Extraction

We analyzed differences in the ex-Gaussian parameters between groups of patients with ADHD and control groups. These differences are usually analyzed in the primary studies through t-tests or ANOVA tests, from which Cohen’s d can be calculated as an effect size. Unstandardized effect size measures may seem appropriate for this meta-analysis since the ex-Gaussian parameters from RT distributions usually share the same unit of measurement (milliseconds or, less frequently, seconds). Nonetheless, magnitudes vary widely across tasks and samples. *µ* varies from 200 to almost 1000 ms, *σ* varies from 15 to 250 ms, and *τ* varies from 30 to 400 ms. This heterogeneity in scale measures makes using standardized effect size measures more suitable (Cheung & Vijayakumar, 2016). Positive d values indicate a greater parameter value for the ADHD sample, and conversely, negative d values indicate a greater parameter value for the control sample.

Using Cohen’s d as an effect size requires the sample distributions of variables to be normal. *µ* being the mean of the Gaussian part of the distribution, it is clear that *µ* is theoretically normal. On the contrary, *σ* and *τ* are not normally distributed (Gmehlin et al., 2014; Leth-Steensen et al., 2000); rather, the sample means of *σ* and *τ* are gamma distributed. In fact, *σ* fits a chi-square distribution, which is a particular case of a gamma distribution. Gamma distributions are a good approximation for Gaussian distributions with sample sizes larger than 30 (Johnson & Kotz, 1970). For this reason, we included in analysis for *σ* and *τ* only those studies with sample sizes of 30 or more participants on each group.

For every study, if Cohen’s d values were provided, they were directly extracted. If the mean and standard deviation (or analogous measurements like standard error for means) were provided, Cohen’s d was calculated through Psychometrica (Lenhard & Lenhard, 2016). If statistical tests and sample sizes were provided, Cohen’s d values were calculated. Otherwise, if *η*^*2*^ values were provided, Cohen’s d values were calculated following Cohen (1988). In these cases, the effect direction was determined observing which sample mean was larger. The effect was positive if the ADHD sample had a larger mean, and negative if the control group had a larger mean. If none of the above were provided, corresponding authors were contacted for further data.

Five studies (Duffy et al., 2021; Halliday et al., 2021; Keith et al., 2017; Thissen et al., 2014; Zhao et al., 2021) only reported analyses for *τ*. Four other studies (Borella et al., 2013; Gmehlin et al., 2016; Osmon et al., 2018; Rosch et al., 2013) reported non-significant effects for *µ*, without providing further data. In these cases, as recommended in Rosenthal (1995), they were assigned effect size values to 0, rather than excluding the study and thus incrementing the bias risk. We performed a sensitivity analysis to study what would have happened with the estimation of *µ* effect had these studies been excluded (see Appendix 1). In summary, retaining these four studies had little effect on the estimation of *µ* effect or the risk of bias, as seen in Figure S1.

In every case, intra-study variances were calculated from Cohen’s d and sample sizes through the corresponding equation.

### Data Analysis

All the analyses were performed through the Metafor package for R (Viechtbauer, 2010). First, we grouped outcomes in clusters according to the following rules. Two outcomes from the same study were assigned to the same cluster, and outcomes from two different studies which shared at least one author were also assigned to the same cluster, unless the authors declared that there was no overlapping between samples, or we could reasonably assume that there was no overlapping between samples. We identified seven clusters of studies sharing one or more authors. In these cases, we contacted the authors to clarify whether there was sample overlap. Three of them declared there was no between-study sample overlapping. Another cluster contained two studies with a large time lapse between them and we decided that there could not have been overlapping between their samples. The three remaining clusters were treated as having overlapping samples. See Appendix 2 for details. Once the clusters were established, three-level models were performed to obtain pooled effect size estimations, with the cluster as a random effect (Assink & Wibbelink, 2016; Van den Noortgate et al., 2015). Overall results were displayed in a forest plot. The same procedure was followed when analyzing subsets of studies or clusters defined by the cognitive domains. Also, three-level mixed-effects models were fitted to explore the relationship between the effect sizes and a group of potentially moderator variables. Pseudo-R^2^ estimations (Cheung, 2015) were performed for each parameter and variance component through the equations, ${R}_{W}^{2}=\frac{{s}_{W:M1}^{2}-{s}_{W:M2}^{2}}{{s}_{W:M1}^{2}}$ [1] and ${R}_{B}^{2}=\frac{{s}_{B:M1}^{2}-{s}_{B:M2}^{2}}{{s}_{B:M1}^{2}}$ [2], where R^2^_W_ and R^2^_B_ are the pseudo-R^2^ for within-cluster and between-cluster components, s^2^_W:M1_ and s^2^_B1_ are the within-cluster variance component of the model without moderator, and s^2^_W:M2_ and s^2^_B:M2_ are the within-cluster and between-cluster variance components of the model with moderator. Negative values for pseudo-R^2^ were set to 0.

Heterogeneity between studies was assessed through the within-cluster and between-cluster components of variance. The significance of each variance component was determined by performing log-likelihood-ratio tests comparing the variances of the full models with the variances of models in which the within-cluster or between-cluster variances, respectively, were fixed to 0 (Assink & Wibbelink, 2016).

Regarding potential publication bias, regression tests, trim-and-fill methods, and estimation of fail-safe numbers are the most common methods for bias assessment. Nonetheless, when outcomes are not independent, some of these methods are discouraged. For this reason, following recommendations from Nakagawa et al. (2022), we performed funnel plots and tested their significance through multi-level meta-regression models using sample size as a fixed-effect moderator variable. Using sample sizes seems to perform better than using standard errors with non-independent outcomes (Fernández-Castilla et al., 2021; Nakagawa et al., 2022). More specifically, these authors suggest using the inverse of the “effective sample size” instead of the sum of the sample sizes. The effective sample size is calculated as follows: ${{\rm{\tilde n}}_i} = {{{n_{1i}}{n_{2i}}} \over {{n_{1i}} + {n_{2i}}}}$, where ñ_i_ is the effective set size of study i, n_1i_ is the sample size of the group 1 in study i, and n_2i_ is the sample size of the group 2 in study i. We first used the square root of the inverse of the effective set size, $\sqrt {\frac{1}{{\widetilde {{n_i}}}}} = \sqrt {\frac{{{n_{1i}} + {n_{2i}}}}{{{n_{1i}}{n_{2i}}}}} = \sqrt {\frac{1}{{{n_{1i}}}} + \frac{1}{{{n_{2i}}}}}$, to test the significance of funnel asymmetries.

## Results

### Overall Results

**Fig. 2:**
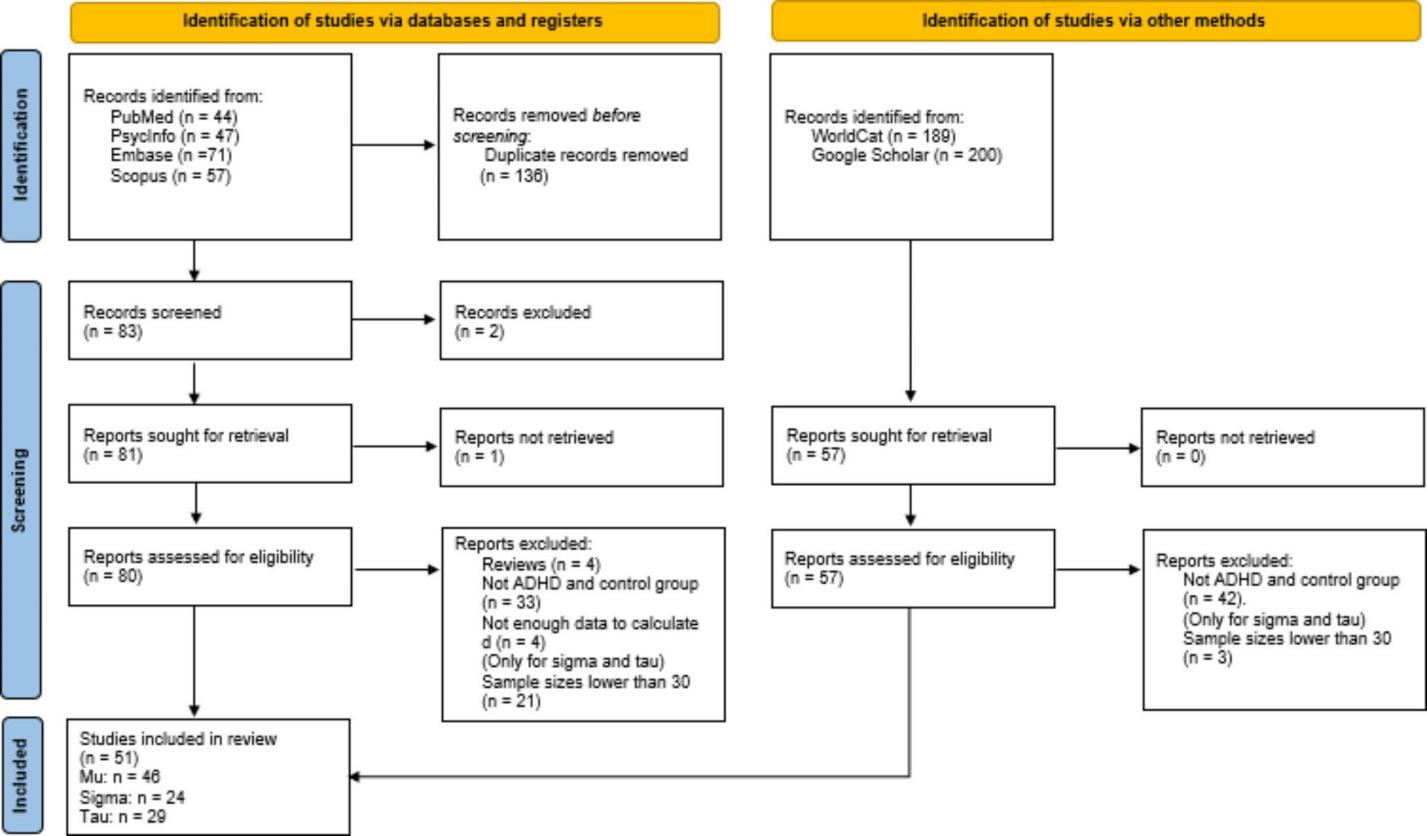
Flow diagram of searches for this meta-analysis

A total of 51 studies were included in the present meta-analysis. The flow diagram is presented in Fig. 2.

**Fig. 3:**
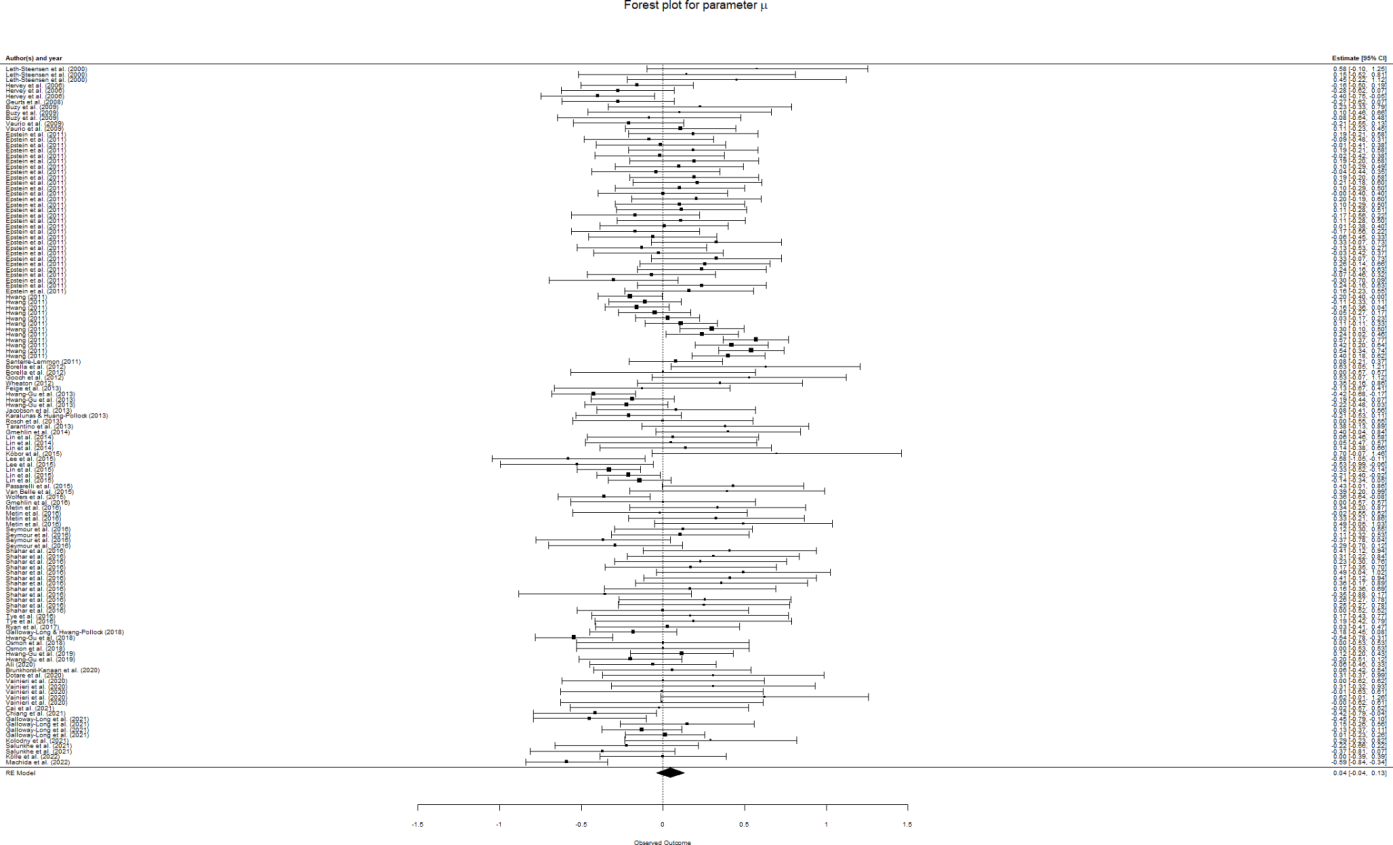
Forest plots for parameter *µ*. Parameter *µ* shows no global differences between ADHD patients and healthy controls

**Fig. 4:**
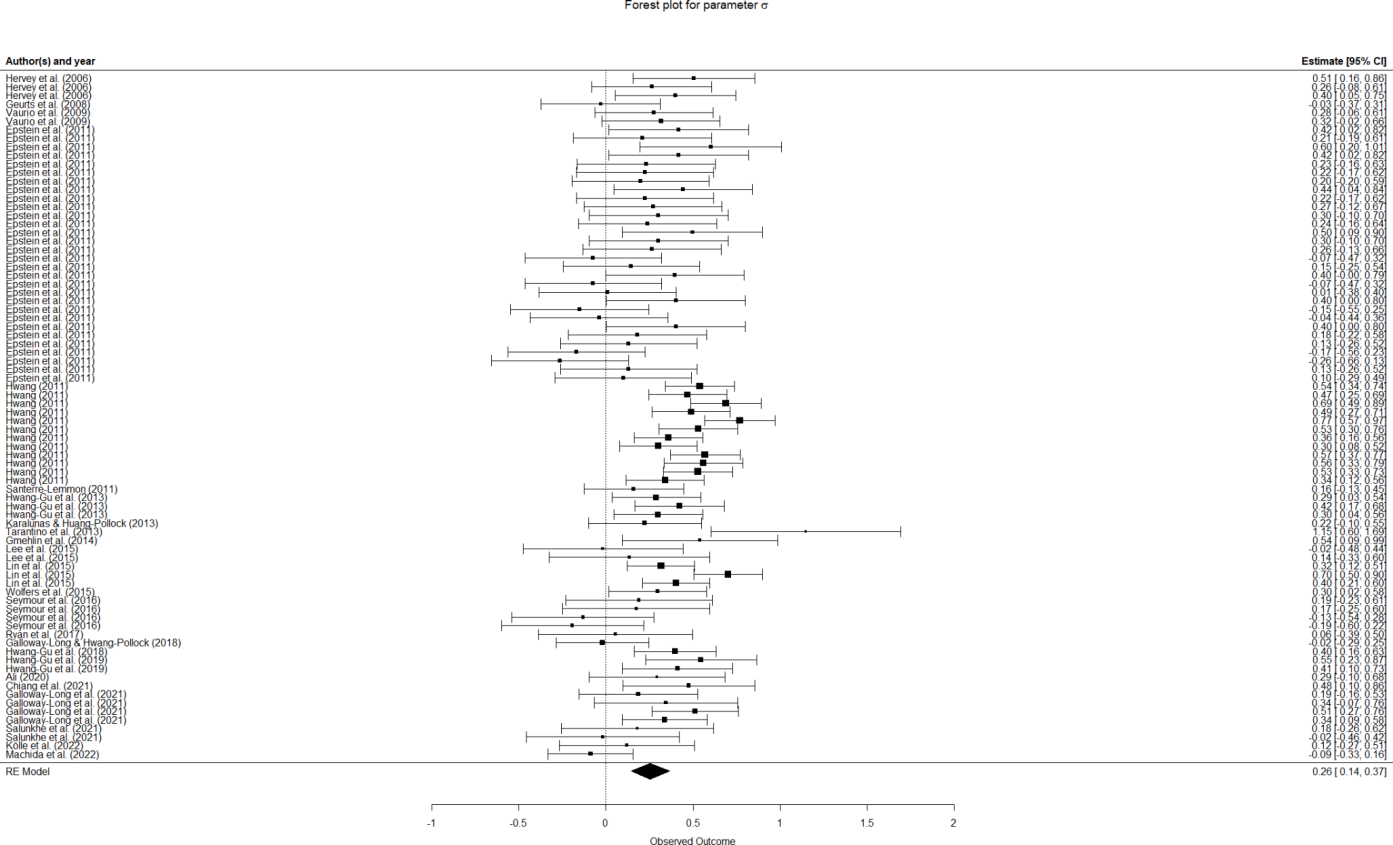
Forest plot for parameter *σ*

**Fig. 5:**
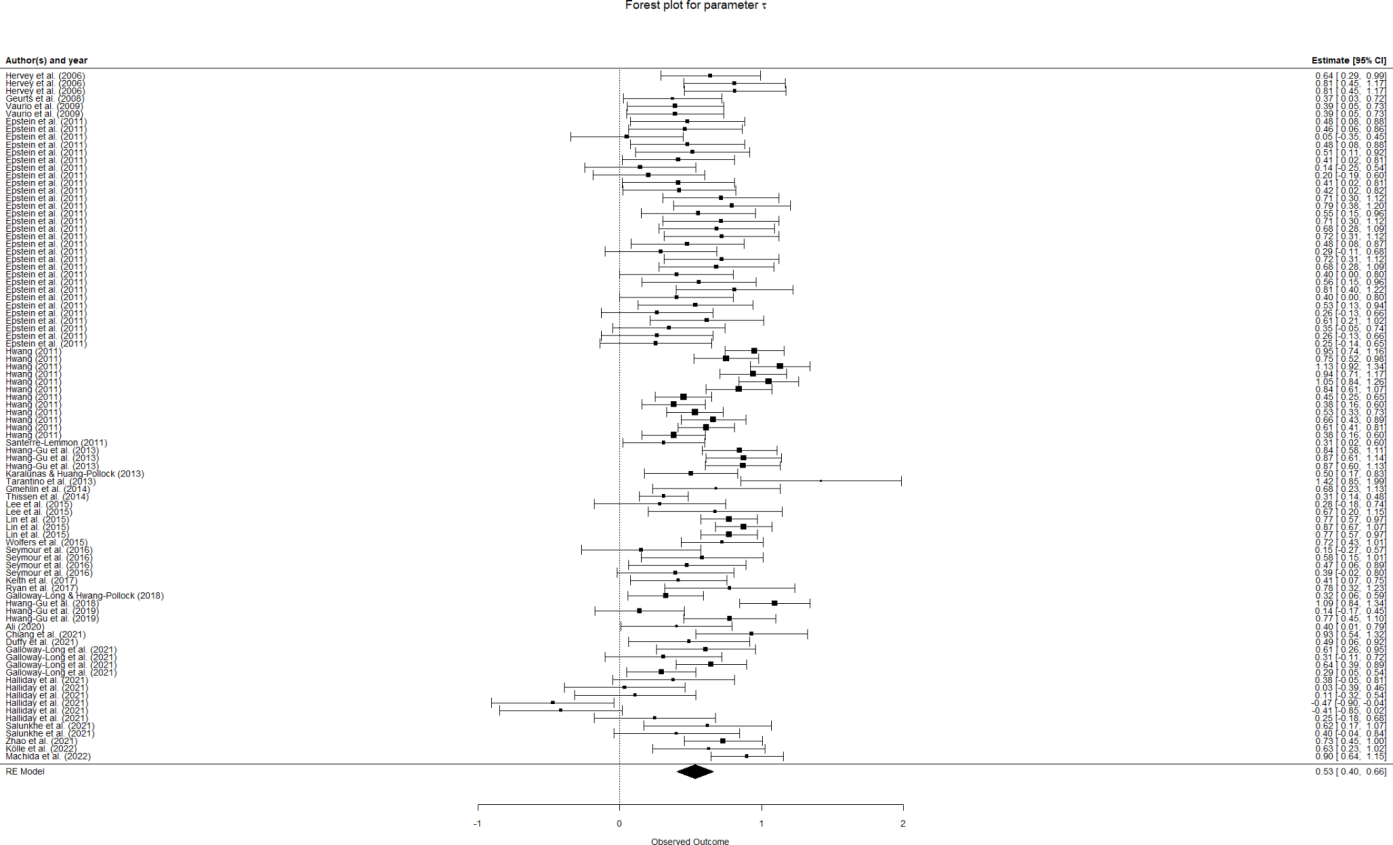
Forest plot for parameter *τ*

The overall results for this meta-analysis are summarized in the forest plots in Fig. 3 (*µ*), Fig. 4 (*σ*), and Fig. 5 (*τ*). The pooled effect for *µ* was *d* = 0.0447 (95% CI: [-0.0399,0.1292]). Thus, our meta-analysis found no overall differences in *µ* value between ADHD and control groups. The overall differences were found to be moderate for *σ* (*d* = 0.2551, 95% CI: [0.1441,0.3660]) and moderate-to-large for *τ* (*d* = 0.5294, 95% CI: [0.3992,0.6597]), according to the usual interpretation for Cohen’s d (Cohen, 1988). Heterogeneity was estimated to be statistically significant for all parameters and variance components, except for the between-cluster variance of *µ* (Table 1), which confirmed the convenience of estimating a random effects model for the three parameters, as well as indicating that a number of variables may moderate the differences for the three ex-Gaussian parameters.

**Table 1:** Within-cluster and between-cluster heterogeneity estimations for the ex-Gaussian parameters

	Within-cluster variance	Significance	Between-cluster variance	Significance
*µ*	0.0359	< 0.0001	0.0182	0.0574
*σ*	0.0077	0.0346	0.0247	< 0.0001
*τ*	0.0246	< 0.0001	0.0447	< 0.0001

### Analysis of Moderator Variables

#### ADHD Subtype

Twenty of the included studies differentiated between three ADHD subtypes, including predominantly hyperactive, predominantly inattentive, and mixed or combined. These three subtypes differ in terms of their symptomatology and thus may present differences in cognitive tasks. Only one of these studies (Epstein et al., 2011) explicitly used the ADHD subtype as a factor to explore differences in ex-Gaussian parameters, but the other studies differed in the reported proportion of patients with each ADHD subtype. We coded these reported proportions and performed a meta-regression analysis. Of course, for every primary study, the proportions of patients for each ADHD subtype are not independent variables, rather, one of the proportions is a linear combination of the two others. Thus, we estimated meta-regression models for the three parameters taking the proportion of patients with the hyperactive ADHD subtype and the proportion of patients with the inattentive ADHD subtype as moderator variables.

Meta-regression models did not account for a significant proportion of variability for *µ* (QM(2) = 0.8802, p = .6440, R^2^_W_ = 0.2880, R^2^_B_ = 0). For *σ*, the meta-regression was significant (QM(2) = 7.8783, p = .0195,, R^2^_W_ = 0.6472, R^2^_B_ = 0) and the meta-regression coefficient for the proportion of inattentive subtype was significant (β = -0.1397, p = .0064). For *τ* the meta-regression was not significant (QM(2) = 3.3368, p = .1885,, R^2^_W_ = 0, R^2^_B_ = 0), but the coefficient for inattentive subtype approached significance (β = -0.1307, p = .0679). The meta-regression coefficients suggest that, the larger the proportion of inattentive ADHD patients in the clinical sample, the shorter the differences in parameter *σ* (and perhaps *τ*) between clinical and typical samples.

#### Task or Cognitive Domains Assessed

Tasks were then classified into cognitive domains according to the classification used in Ging-Jehli et al. (2021), which specifies six domains for the most common cognitive tasks, namely, cognitive flexibility, selective attention, working memory, time perception, sustained attention, and inhibitory control. Every task was assigned to one or more cognitive domains. As observed in Castellanos et al. (2006), “hot” executive functions, rather than “cool” functions, were also predominantly observed in the selected studies. Table 2 summarizes how the tasks used in the primary studies were classified.

**Table 2:** Classification of tasks used in the included studies by cognitive domain (Ging-Jehli et al., 2021). It is noteworthy that some cognitive domains outnumber the total reviewed studies because several articles provided results for more than one experimental condition

Cognitive domain	Tasks included	Outcomes for every cognitive domain		
		*µ*	*σ*	*τ*
Cognitive flexibility	Posner task Flanker tasks Stroop tasks Change task	9	1	1
Selective Attention	Attentional Network task Flanker tasks Stroop tasks Posner task	15	6	6
Working memory	n-back task Classification tasks Visual Serial Addition Task	24	8	8
Time Perception	Motor Timing task	6	6	7
Sustained attention	Simple reaction time tasks Warned reaction time task Fast task Go task Go/No go Oddball task Sustained Attention to Response task WAF – Vienna Test Systems Rapid Automatized Naming task Choice tasks Finger tapping task	73	49	54
Inhibitory control	Go/No go Stop signal Change task Visual inspection time task Stroop tasks Continuous Performance Tests Jelly bean task Multi-source interference task	66	51	59

We then generated six new dichotomous variables, one for each cognitive domain. Thus, for every study and task these variables accounted for whether or not the domains were measured. After aggregating outcomes by clusters, random effect models were estimated for each cognitive domain separately, in order to obtain effect size estimations for the differences between ADHD patients and healthy controls for every cognitive domain. These estimations are provided in Table 3.

**Table 3:** Estimated effect sizes and CI (95%) for *µ*, *σ*, and *τ* for cognitive domains. Significant differences are in bold characters

	Cognitive Flexibility	Inhibitory Control	Sustained Attention	Selective Attention	Working Memory	Time Perception
*µ*	0.19 [-0.05, 0.43]	0.01 [-0.13, 0.14]	-0.02 [-0.13, 0.10]	**0.20** [0.08, 0.33]	0.05 [-0.11, 0.20]	**0.42** [0.31, 0.52]
*σ*	-0.03^*^ [-0.37, 0.31]	**0.27** [0.14, 0.41]	**0.31** [0.17, 0.44]	**0.23** [0.07, 0.39]	0.15 [-0.03, 0.33]	**0.45** [0.35,0.54]
*τ*	**0.37** ^*^ [0.03, 0.72]	**0.53** [0.36, 0.60]	**0.62** [0.49, 0.76]	**0.49** [0.33, 0.66]	**0.46** [0.33, 0.60]	**0.42** [0.24, 0.61]

^*^The estimation is based on only one outcome.

For *µ*, only effect sizes for selective attention and time perception tasks were non-zero. For tasks involving selective attention, people with ADHD tended to show larger *µ* values. Also, differences (non-overlapping CI for effect sizes) were observed between time perception and working memory. In tasks involving selective attention and time perception, participants with ADHD tended to respond more slowly than controls.

For *σ*, estimated effect sizes for all cognitive domains except working memory and cognitive flexibility were significant and positive, which means that *σ* parameter tended to be larger for ADHD patients than for controls. These differences were larger for time perception and selective attention, and smaller for inhibitory control, sustained attention, and working memory.

For *τ*, estimated effect sizes for all cognitive domains were positive. As expected, the *τ* parameter also tended to be larger for people with ADHD than controls. The *τ* effect was slightly variable across cognitive domains, with sustained attention and inhibitory control being the domains where the effects were larger, and cognitive flexibility the domain where the effect was smaller.

#### Age

For every study, mean age for the ADHD and control group was recorded. We calculated the weighted mean of ages from the two groups to obtain the total sample mean age.

We fitted a mixed-effects model for each ex-Gaussian parameter with the sample mean age as a moderator variable. Regression models were not significant for *µ* (QM(1) = 0.5429, p = .4612, R^2^_W_ = 0, R^2^_B_ = 0.1648), *σ* (QM(1) = 0.0127, p = .9102, R^2^_W_ = 0, R^2^_B_ = 0) or *τ* (QM(1) = 2.2164, p = .1365, R^2^_W_ = 0.0304, R^2^_B_ = 0). Differences in ex-Gaussian parameters between people with and without ADHD seem to remain approximately constant across ages.

#### Gender

Forty-seven studies reported the proportions of males and females in the experimental groups. The vast majority of studies reported a larger proportion of females in control groups than ADHD groups. The proportion of females in ADHD and control groups were found to be highly correlated (Spearman’s ρ = 0.870, p < .001) and thus the two moderators combined were not suitable for meta-regression. Thus, we fitted meta-regression models using only the proportion of females in ADHD groups. For these meta-regression models, positive values indicate larger female proportions.

The effect of gender proportion was not significant for *µ* (QM(1) = 0.4499, p = .5024, R^2^_W_ = 0.0026, R^2^_B_ = 0.0482), *σ* (QM(1) = 0.2640, p = .6074, R^2^_W_ = 0, R^2^_B_ = 0) or *τ* (QM(1) = 0.7971, p = .3720, R^2^_W_ = 0, R^2^_B_ = 0).

Also, the difference between female proportions was calculated for each study as follows. $\varDelta \left(\% Female\right)={\% Female}_{Control}- {\% Female}_{ADHD}.$ This difference was used as a moderator variable for meta-regression models. Positive values indicate a larger proportion of females in the control group. For *µ* and *σ*, the meta-regression model with the proportion difference as a predictor variable accounted for a non-significant amount of variability, and the respective coefficients were not significant for *σ* (QM(1) = 2.4014, p = .1212). The regression coefficients were significant for *µ* (QM(1) = 9.1929, β = 0.0094, p = .0024) and *τ* (QM(1) = 5.9540, β = -0.0083, p = .0147).

#### Interstimulus Interval (ISI)

In 21 studies, mostly using Go/No Go and Continuous Performance Tests (CPT), interstimulus intervals (ISI) were reported. We estimated meta-regression models with the ISI as a predictor variable. The coefficient of linear regression approached significance for *σ* (β = -0.0392, p = .0505, R^2^_W_ = 0, R^2^_B_ = 0), but was not significant for *µ* (p = .0970, R^2^_W_ = 0.9421, R^2^_B_ = 0) or *τ* (p = .2965, R^2^_W_ = 0.5823, R^2^_B_ = 0.2574).

**Fig. 6:**
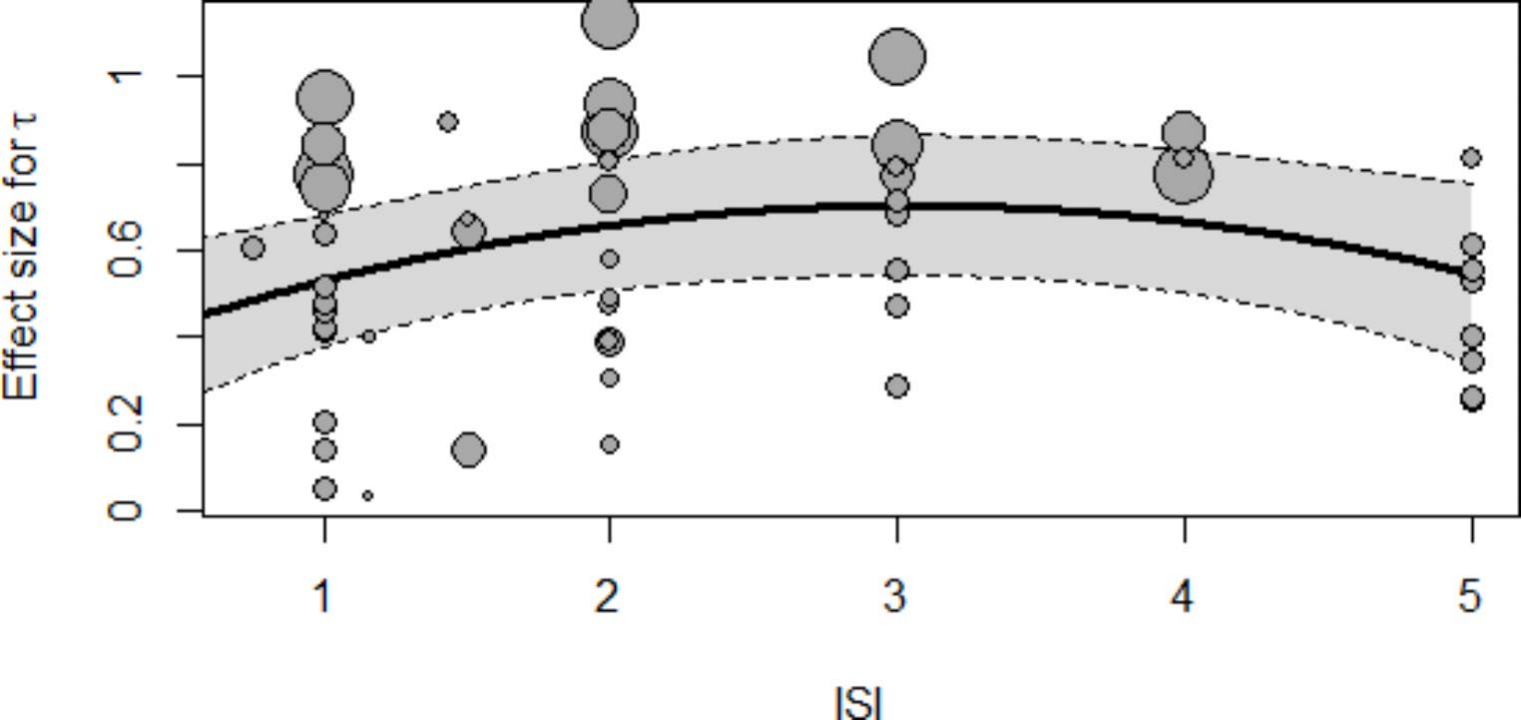
Meta-regression curve for *τ*. The predictor variable is ISI. The quadratic relationship between ISI and *τ* somehow resembles the one predicted by the State Regulation hypothesis (Metin et al., 2016)

We also estimated quadratic meta-regression models for the three parameters. The non-intercept coefficients of *µ* and *σ* were non-significant in all these models (all p > .1 except the second-grade coefficient for sigma, β_2_ = -0.0293, p = .0607), but the coefficients associated with the quadratic model on *τ* were statistically significant (β_1_ = -0.2655, p = .0042; β_2_ = -0.0428, p = .0075). Figure 6 shows the meta-regression lines.

These results suggest that ISI does not alter the difference on the usual reaction speed, but does alter the difference on intertrial variability. On one side, as ISI increases, differences in intertrial variability of usual responses tend to mitigate. On the other side, there seems to be an optimum of differences in reaction time lapses at around three seconds of ISI.

#### Intelligence Quotient (IQ)

**Fig. 7:**
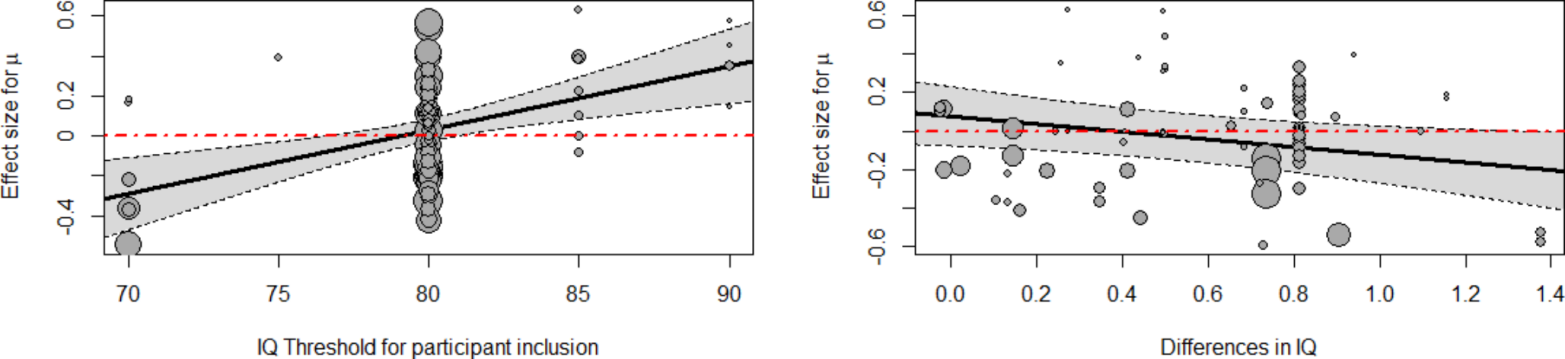
Meta-regression plots for *µ* as a function of IQ threshold of exclusion criterion and differences in IQ between samples, respectively. An IQ threshold of 80 is the most widely used. Thresholds below 80 tend to result in faster reactions from patients with ADHD and thresholds above 80 tend to result in faster responses from people without ADHD. Regarding differences in IQ, only differences below 0.4 IQ points are related with slower reactions from people with ADHD, while, as these differences become larger, the groups of patients with ADHD tend to respond faster than groups of people without ADHD.

A total of 35 studies reported sample IQs, and 28 of these studies excluded participants with IQ below a certain threshold. We compared the estimations of studies with and without this IQ-based exclusion criterion. The existence of an IQ threshold did not result in a significant difference for any parameter (*µ*: QM(1) = 0.1423, p = .7060, R^2^_W_ = 0.0595, R^2^_B_ = 1; *σ*: QM(1) = 0.1328, p = .7156, R^2^_W_ = 0.0283, R^2^_B_ = 0; *τ*: QM(1) = 0.0001, p = .9917, R^2^_W_ = 0, R^2^_B_ = 0.4711). However, among those studies which did apply an IQ threshold, there was a significant effect of the IQ threshold value on the effect of *µ* (QM(1) = 12.8246, β = 0.0319, p = .0003, R^2^_W_ = 0.0085, R^2^_B_ = 0), but not *σ* (QM(1) = 1.5757, p = .2094, R^2^_W_ = 0.0120, R^2^_B_ = 0) or *τ* (QM(1) = 0.0361, p = .8492, R^2^_W_ = 0, R^2^_B_ = 0). Figure 7 shows the meta-regression for *µ*, and shows how a threshold of 80 seems to separate positive and negative differences in *µ*.

We also analyzed a possible effect of the difference in IQ between participants with and without ADHD. No significant results were obtained for *σ* or *τ* (*σ*: QM(1) = 0.1629, p = .6865, R^2^_W_ = 0, R^2^_B_ = 0.1105; *τ*: QM(1) = 0.4766, p = .4900, R^2^_W_ = 0.5746, R^2^_B_ = 0.3230), but *µ* showed a negative significant effect (QM(1) = 4.8373, β = -0.1996, p = .0279, R^2^_W_ = 0.9886, R^2^_B_ = 0), as described in Fig. 7.

#### Stimulant Medication use

Regarding medication use, 39 studies asked those participants with ADHD who were being treated with stimulants to discontinue this treatment 24 or 48 h prior to participating in the studies. Another five studies directly excluded those participants with ADHD who were taking stimulants. The rest of the studies did not apply any rules regarding medication use. It is possible that these different manners to deal with medication use among the samples had an effect on the estimations. To explore this possibility, we compared the estimations of these three study subgroups, using the medication modality as a moderator variable. We did not obtain significant differences in *µ* (QM(2) = 2.8482, p = .2407, R^2^_W_ = 0, R^2^_B_ = 0.1974), *σ* (QM(2) = 3.2372, p = .1982, R^2^_W_ = 0, R^2^_B_ = 0) or *τ* (QM(2) = 4.1879, p = .1232, R^2^_W_ = 0.0562, R^2^_B_ = 0).

#### Comorbidities

A large number of comorbidity-related exclusion criteria were used in one or more of the included studies. Among those exclusion criteria, we selected the more frequent or more commonly related with ADHD to test whether adding these exclusion criteria could affect the differences on parameters. These results are summarized in Table 4.

**Table 4:** Moderator analysis using the presence or absence of comorbidities as exclusion criteria

	Neurological disorders		Mood disorders		Anxiety disorders		Autism Spectrum Disorders		Schizophrenia or Psychosis		Sensory or motor impairments		Obsessive Compulsive Disorder	
	QM(1)	P value	QM(1)	P value	QM(1)	P value	QM(1)	P value	QM(1)	P value	QM(1)	P value	QM(1)	P value
*µ*	**7.5223**	**0.0061**	0.7786	0.3776	2.3781	0.1230	**8.6614**	**0.0033**	0.8338	0.3612	1.0638	0.3024	0.0317	0.8588
*σ*	3.0585	0.6079	0.7090	0.3998	0.6507	0.4199	0.3752	0.5402	**5.1457**	**0.0233**	2.2906	0.1302	0.8652	0.3523
*τ*	0.0165	0.8978	0.4191	0.5174	0.6519	0.4194	0.8338	0.3612	3.2620	0.0709	0.4414	0.5064	0.4286	0.5127

Only excluding patients with schizophrenia or psychosis had a statistically significant impact on *σ* estimation. The *σ* effect estimated for studies excluding patients with schizophrenia or psychosis was 0.4058 (95% CI: [0.2923, 0.5192] and 0.2120 (95%CI: [0.0752, 0.3489]) for studies not excluding these patients. Excluding patients with schizophrenia or psychosis reduced the variability in usual reactions. Also, excluding patients with neurological disorders or autism spectrum disorders (ASD) had a significant impact on *µ* estimations. The *µ* effect estimated on studies excluding patients with neurological disorders was 0.0575 (95% CI: [-0.0477, 0.1626] and − 0.0230 (95%CI: [-0.1600, 0.1140]) on studies not excluding them. In turn, the *µ* effect estimated on studies excluding patients with ASD was − 0.0578 (95% CI: [-0.2085, 0.0929] and 0.0556 (95%CI: [-0.0400, 0.1512]) on studies not excluding these patients. Excluding patients with neurological disorders and with ASD seemed to have opposite effects. Excluding patients with neurological disorders made the ADHD group faster than the control group, while excluding patients with ASD made the ADHD group slower than the control group.

### Risk of Bias

As described in the Methods section, publication bias was assessed through funnel plots and meta-regression models using effective sample size as a moderator variable.

Funnel plot asymmetry was statistically significant for *µ* (QM(1) = 10.4859, p = .0012) and was statistically non-significant for *σ* (QM(1) = 0.8617, p = .3533) and *τ* (QM(1) = 0.5104, p = .4750). Parameter *τ* received the most attention in ADHD research, to the point that 10 out of 139 outcomes (7.19%) only reported estimations for *τ*. It is probable that publication bias can be observed in *τ* parameter.

**Fig. 8:**
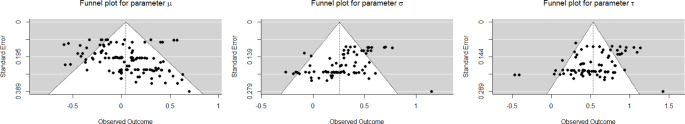
Funnel plots for asymmetry and risk of bias

Funnel plots in Fig. 8 show a small-study effect for *µ*, in the sense that effects tended to be positive for smaller studies, approximately zero for medium-sized studies, and negative for larger studies. Only larger studies tended to show statistically significant results, which suggests publication bias is not mediating significance.

Clearly, the meta-analysis is not biased for parameters *σ* and *τ*. On the contrary, estimation for parameter *µ* is strongly biased to the right. In this case, an overall estimate can be obtained through a meta-regression model with the inverse of the effective sample size, ${1 \over {{{{\rm{\tilde n}}}_i}}}$, as a fixed effect. In our case, this regression model for *µ* resulted in an overall estimation of -0.1724 [-0.3014,-0.0434].

### RT Means and Standard Deviations

Lastly, we analyzed the differences in RT means and standard deviations between people with and without ADHD from the studies that reported these data. The effect size for the differences in RT mean was 0.4213 (95% CI: [0.2759,0.5668]), while for RT standard deviations the difference was 0.6025 (95% CI: [0.4886,0.7164]). These results suggest that patients with ADHD tend to have RTs with larger means and deviations. Also, both RT mean and standard deviation may discriminate between people with and without ADHD.

## Discussion

Overall, we can conclude that the “*τ* effect”, as named by Leth-Steensen et al. (2000), as well as its homologous “*σ* effect”, are generally confirmed. *τ* effect is greater than *σ* effect. Studies combined show a lack of evidence for a “*µ* effect”, but this finding is biased to some extent. RT analysis based on ex-Gaussian parameters has been proposed to discriminate between individuals with and without ADHD for classification purposes (Galloway-Long et al., 2021; Hernaiz-Guijarro et al., 2019; Leth-Steensen et al., 2000; Wheaton, 2012), but a lack of discrimination power has also been reported (Brunkhorst-Kanaan et al., 2020). Our findings are support the use of either intertrial variability parameters, *σ* or *τ*, for screening purposes. Our results also suggest that parameter *µ*, the central tendency of regular responses, is generally not discriminative.

In general, high levels of *σ* and *τ* may indicate the presence of ADHD symptomatology. This finding supports the use of intertrial variability for detecting ADHD, and challenges the use of usual descriptive statistics (i.e., mean and standard deviation). Also, differences in RT means and standard deviations have the same direction (larger for patients with ADHD) and similar effect sizes. These results would suggest that the RTs of patients with ADHD are larger and more variable. Nonetheless, our analysis with ex-Gaussian parameters only suggest larger intertrial variability, without clear differences in speed. In fact, regarding *µ*, although our meta-analysis shows a general lack of difference between patients with ADHD and controls, unbiased results point towards a slight general *µ* effect in the inverse direction than the two other effects, that is, ADHD samples tend to show lower values for *µ*.

### Cognitive Domains

Our analysis for cognitive domains revealed that tasks involving selective attention and time perception show effects on the three ex-Gaussian parameters. Impaired selective attention or, more specifically, increased distractibility, is a characteristic of patients with ADHD (Ging-Jehli et al., 2021). Our results indicate that patients with ADHD tend to be slower than people without ADHD in these tasks, and suggest using RTs in these kinds of tasks to discriminate between people with and without ADHD, but our results do not reveal the origin of this increased reaction time. Particularly noteworthy are the results for time perception, which show moderate effects for the three parameters. Two recent reviews (Nejati & Yazdani, 2020; Ptacek et al., 2019) show that impairments in time perception in patients with ADHD are significant and present, although with varying intensity, across time-related task types (time discrimination or estimation) and sensorial modalities. Ptacek et al. (2019) even proposed time perception impairment as a diagnostic criterion for ADHD. Our results are consistent with this proposal. Sustained attention and, in a less clear manner, inhibitory control may also discriminate between people with and without ADHD, but only taking into account the intertrial RT variability. On the other hand, tasks involving working memory and cognitive flexibility only show an effect for *τ*. It is worth noting that only one outcome was included in the analysis of *σ* and *τ* effect on cognitive flexibility. Further research is needed to better establish the characteristics of RT distributions of people with ADHD in tasks involving cognitive flexibility. Although there is evidence that working memory is impaired in patients with ADHD (Alderson et al., 2013; Kasper et al., 2012), our results only support detection of working memory impairment through increased *τ*.

### Age and Gender

Our results suggest that differences in intra-individual variability (*σ* and *τ*) is significant between ADHD and control groups, and that these differences seem to remain constant across developmental periods. This result is consistent with the previous finding that intra-individual variability in people with ADHD seems to be more similar to younger controls than to age-matched controls (Leth-Steensen et al., 2000; van Belle, van Hulst, et al., 2015), showing a possible developmental impairment in inter-trial variability in ADHD samples. Consistently, Castellanos et al. (2006) found that patients with ADHD have a less mature cerebral cortex. Also, Van Belle, van Raalten, et al. (2015) showed that intra-individual variability in people with and without ADHD is mediated by different brain regions in different developmental phases, which could explain the impairment in intra-individual improvements.

Our results also suggest that differences in *µ* and *σ* remain constant regardless of the gender proportions or the differences in the gender proportions between ADHD and control groups. On the contrary, differences in *τ* become larger as the differences in the proportions of females decrease (and the differences in the proportions of male increase). Conversely, for parameter *µ*, the effect is the opposite. The larger the differences in the proportions of males between ADHD and control groups, the larger the effect of *µ*. Several reviews found evidence of greater impulsivity (Gershon, 2002; Hasson & Fine, 2012), hyperactivity (Gaub & Carlson, 1997; Gershon, 2002), and inattention (Gaub & Carlson, 1997; Gershon, 2002) among male patients compared to female patients. Hasson and Fine (2012) found that the difference between males with and without ADHD in impulsivity measures from the CPT test was larger than the difference between females with and without ADHD. Taken together, this evidence supports the interpretation of parameters *µ* and *τ* in terms of impulsivity or inattention.

### ISI

ISI was hypothesized to influence the differences in RTs between patients with ADHD and controls (Hwang-Gu, Chen, et al., 2019; Hwang-Gu et al., 2013; Lee et al., 2015; Leth-Steensen et al., 2000; Metin et al., 2016). In particular, Hwang-Gu et al. (2013, 2019a, b), based on results from Leth-Steensen et al. (2000), hypothesized a direct relationship between *τ* and ISI, as an effect of increasing cognitive load with increased ISI. Metin et al. (2016) hypothesized a quadratic (U-shaped) relationship between *σ* and ISI. Both linear and quadratic coefficients in regression with *σ* approached significance, which could be taken as evidence supporting a relationship between *σ* and ISI, as hypothesized (Metin et al., 2016). This quadratic relationship is more evident for *τ*. The sample sizes used in some of these studies may have contributed to a premature confirmation of the hypotheses, in particular those referred to *σ* and *τ*. It is worth noting that the differential effect of ISI in RT variability is reduced when the ISIs are randomized (“jittered”) in a certain task (Lee et al., 2015; Ryan et al., 2010). It is possible that jittering ISIs reduces boredom in ADHD patients. Also, it is possible that predictable ISIs allow people with ADHD some sort of planning on attentional lapses.

### IQ

In general, our results do not support any relationship between IQ and differences in intertrial RT variability. We did find that, although imposing a lower threshold on the IQ of participants does not significantly affect the results, researchers should be careful if they do so. Taking into account only studies with a lower threshold for IQ, we found that a low IQ threshold may influence differences in usual RTs. Starting from 80 IQ points, the upper the threshold, the larger the differences between people with and without ADHD. Interestingly, including patients with an IQ below 80 results in negative differences in *µ*, which indicates that groups of patients with ADHD tend to respond faster than groups of patients without ADHD.

The difference in IQ may also have an effect on differences in *µ*. Our results suggest that when samples have similar IQ people with ADHD tend to respond slower than people without ADHD and, when these differences are larger, people with ADHD tend to respond faster than people without ADHD. In the context of intelligence and RT, it is generally accepted that people with higher IQs tend to respond faster (Der & Deary, 2003; Matthews & Dorn, 1989; Ratcliff et al., 2008). Also, in almost all studies included in this meta-analysis, samples of participants without ADHD had a higher mean IQ than samples of patients with ADHD. To summarize, we would expect that larger differences in IQ lead to larger, positive differences in *µ*, contrary to what we found.

### Medication and Comorbidities

Our results show that medication status had little impact on the differential performance of people with and without ADHD. This may indicate that people participating in this kind of experiment are generally following long-term therapies, and withdrawing medication has little effect in performance on cognitive tasks. Also, we found effects of excluding patients with schizophrenia, neurological disorders, and ASD. Excluding patients with schizophrenia or psychosis decreased the difference in intra-individual RT variability, consistent with literature suggesting a relationship between schizophrenia and increased intra-individual RT variability (Kaiser et al., 2008; Karantinos et al., 2014; Smyrnis et al., 2009; Vinogradov et al., 1998). When excluding participants with ASD, groups with ADHD tended to be faster than groups without ADHD, which suggested that participants with ASD are slower than control groups. Consistently, Salunkhe et al. (2021) observed that participants with both ADHD and ASD had larger *µ* values than participants with only ADHD and controls. However, the same authors found a similar effect for *σ* and *τ*, which is not consistent with the lack of effect that excluding patients with ASD had in the present meta-analysis. Furthermore, Karalunas et al. (2014) reported increased inter-trial variabilities in RT only when patients with ASD also had ADHD. Last, excluding patients with neurological disorders resulted in negative differences in parameter *µ*, which may suggest that patients with neurological disorders respond faster, perhaps due to increased impulsivity.

### Cognitive Interpretations for Ex-Gaussian Parameters

As pointed out in the introduction, *τ* is the most analyzed parameter in terms of interpretation. It is generally accepted that larger *τ* values denote larger and more frequent RTs in the tail of the distribution. This finding has been interpreted in terms of attentional lapses, impairments in cognitive energy regulation, or impairments in information accumulation speed. Shahar et al. (2016) state that attentional lapses, or “mind-wandering”, should be more or less constant across tasks. According to our results, differences in *τ* are similar across cognitive domains, consistent with the usual interpretation for *τ*. Nonetheless, our analysis on ADHD subtypes suggests that *τ* is inversely related to inattention.

Our results suggest that *σ* differences are estable across age, gender, ISI, and ADHD subtypes. Only four cognitive domains had an influence on the intensity of differences in *σ*, being time perception and selective attention those for which this difference is larger. This finding suggests that *σ* may be related to attentional control, rather than cognitive change, or memory processes. Yamashita et al. (2021) reported correlations between *σ* and errors in general (e.g., commision, omission, mind-wandering measures, etc.), but the principal component analysis performed by Salunkhe et al. (2020) showed that *σ* and errors load on different components. The same authors argued elsewhere (Salunkhe et al., 2019) that *σ* may be related to neuromodulation or neural “noise”.

*µ* is related to reaction speed, which is influenced by impulsivity. Since impulsivity is a core symptom in ADHD, interpreting parameter *µ* in terms of impulsivity is consistent with our finding that *µ*, and thus reaction times, tend to be faster for ADHD groups.

### Clinical Implications

Regardless of the cognitive process, *τ* tends to discriminate between patients with and without ADHD. *σ* is less useful as a discriminative parameter. Regarding *µ*, in tasks involving sustained attention, people with ADHD tend to respond slightly faster than controls, while in tasks involving selective attention, people with ADHD tend to respond slower. Overall, sustained attention seems to be the only cognitive process where the three parameters show differences between people with and without ADHD. Several recent studies (Brunkhorst-Kanaan et al., 2020; Galloway-Long et al., 2021; Machida et al., 2022) directly assessed the discriminative power of the three parameters through ROC curves. *τ* and *σ* were slightly discriminative, while discriminative power for *µ* was more irregular, ranging from moderate to non-discriminative values.

### Strengths and Limitations

Some reviews and meta-analyses have previously been performed on RT comparisons between groups with and without ADHD (Ging-Jehli et al., 2021; Karalunas et al., 2014; Kofler et al., 2013; Salunkhe et al., 2020; Tamm et al., 2012). The present meta-analysis improves previous work in two ways. First, we were able to search more deeply for the possible influence of potentially moderating variables, such as sample ages or cognitive domains. Second, our search was wide enough to almost completely avoid publication bias or other potentially threatening biases, except for parameter *µ*. On the other hand, one of the limitations of this meta-analysis is concurrent with its scope. Analyses of ex-Gaussian parameters, like any other approach using only RT distributions, do not take into account other indices, such as accuracy measures. Acknowledging the theoretical contributions of ex-Gaussian-based analyses and their usefulness in ADHD detection and more, approaches taking into account other performance variables are encouraged. Diffusion models are an instance of these approaches (Ging-Jehli et al., 2021; Huang-Pollock et al., 2017). Their use in various experimental manipulations helped disentangle the cognitive significance of the ex-Gaussian parameters (Matzke & Wagenmakers, 2009). Last, risk of bias assessment indicated a strong small-sample effect on *µ* estimation. This raises concerns about the reliability of findings based on parameter *µ*. Nonetheless, the potential bias direction has been taken into account, as it likely indicates that *µ* is lower for groups of people with ADHD than directly estimated, and this analysis was incorporated to our findings. Finally, despite having highlighted the advantages of using ex-Gaussian parameters, a limitation on the technical applicability of these findings must also be mentioned. To appropriately fit ex-Gaussian distributions, a minimum of 100 trials per participant is necessary. Also, researchers should quantitatively measure the fit of individual RTs to ex-Gaussian models, because some RT distributions may exceptionally resemble Gaussian distribution, which compromises those particular model fits. Furthermore, sample distributions of *σ* and *τ* parameters, which are the most important parameters in ADHD discrimination, are not Normal, and only in relatively large samples (i.e. >30 observers) do these sample distributions approximate to Normal.

## Conclusion

Estimating ex-Gaussian parameters for RTs is a useful tool to understand the different cognitive profiles, and subsequently distinguish between people with and without ADHD. *τ* and *σ* parameters are larger for groups with ADHD, and these parameters are predictably influenced by the cognitive domain of tasks. Differences tend to be stable across ages. Parameter *µ* tends to be similar in both groups, but this similarity seems to be influenced by developmental stage and task.

### Electronic Supplementary Material

Below is the link to the electronic supplementary material.

Supplementary Material 1

Supplementary Material 2

Supplementary Material 3

Supplementary Material 4

Supplementary Material 5

Supplementary Material 6

Supplementary Material 7

Supplementary Material 8
