# Supplementary material for: Differences in Ex-Gaussian Parameters from Response Time Distributions Between Individuals with and Without Attention Deficit/Hyperactivity Disorder: A Meta-analysis
Source: Neuropsychol Rev. 2023 Mar 6;34(1):320–37. doi: 10.1007/s11065-023-09587-2 (PMC10920450; doi:10.1007/s11065-023-09587-2)
Supplement: Supplementary file 2 — Supplementary Material 2 [file 11065_2023_9587_MOESM2_ESM.docx]

Differences in ex-Gaussian parameters from response time distributions between individuals with and without Attention Deficit/Hyperactivity Disorder: a meta-analysis

Marcos Bella-Fernández^1,2,3^, Marina Martín-Moratinos^1,2^, Chao Li^2^, Ping Wang^2^, Hilario Blasco-Fontecilla^1,2,4,5,*^

^1^Hospital Universitario Puerta de Hierro Majadahonda, Spain.

^2^Universidad Autónoma de Madrid, Spain.

^3^Universidad Pontificia de Comillas, Spain.

^4^CIBERSAM Madrid, Spain.

^5^ITA Mental Health, Madrid, Spain.

*Corresponding author: Hilario Blasco-Fontecilla

[hmblasco@yahoo.es](mailto:hmblasco@yahoo.es)

Statements and Declarations

Funding:

H.B.F. is Principal Investigator (PI) and PhD advisor of Industrial Doctorate Contract (Ministry of Science, Universities and Innovation. Community of Madrid Ref. IND2020/BMD-17544). M.M.M. is the recipient of Industrial Doctorate Contract. M.B.F. has a contract associated with the project.

Author Contribution:

Conceptualization: MBF. Data curation: MBF, MMM, PW. Formal Analysis: MBF, CL. Funding acquisition: HBF, MMM. Investigation: MBF, MMM. Methodology: MBF, CL, HBF. Software: MBF, CL. Supervision: HBF. Visualization: MMM, CL. Writing (Original Draft): MBF, MMM. Writing (Review & Editing): CL, PW, HBF. All authors read and agreed to the final version.

Acknowledgments:

We thankfully acknowledge Cristina Escudero, Head Librarian at the Hospital Universitario Puerta de Hierro Majadahonda, for her advice. We also acknowledge David Osmon, Jeffery Epstein, Jonna Kuntsi, Oliver Grimm, Sarah Karalunas, Cynthia Huang-Pollock, Hillary Galloway-Long, and Dennis Gmehlin for kindly providing further data for this meta-analysis, and Lorraine Maw for proofreading labors.

Conflicts of interest:

In the last two years, H.B.F. has received lecture fees from Takeda. M.M.M. and H.B.F. had recently being granted with the Shibuya Prize (2^nd^ Edition) by Takeda. The rest of the authors declare no conflicts of interests.

Ethical statement: Not applicable.
