# Supplementary material for: Differences in Ex-Gaussian Parameters from Response Time Distributions Between Individuals with and Without Attention Deficit/Hyperactivity Disorder: A Meta-analysis
Source: Neuropsychol Rev. 2023 Mar 6;34(1):320–37. doi: 10.1007/s11065-023-09587-2 (PMC10920450; doi:10.1007/s11065-023-09587-2)
Supplement: Supplementary file 3 — Supplementary Material 3 [file 11065_2023_9587_MOESM3_ESM.docx]

Attention Deficit/Hyperactivity Disorder (ADHD) is one of the most prevalent neurodevelopmental disorders in childhood and adolescence. Differences in Reaction Times (RT) in cognitive tasks have been consistently observed between ADHD and typical participants. Instead of estimating means and standard deviations, fitting non-symmetrical distributions like the ex-Gaussian, characterized for three parameters (µ, σ, and τ), account for the whole RT distributions. A meta-analysis is performed with all the available literature using ex-Gaussian distributions for comparisons between individuals with ADHD and controls. Results show that τ and σ are generally greater for ADHD samples, while µ tends to be larger for typical groups only for younger ages. Differences in τ are also moderated by ADHD subtypes. τ and σ show, respectively, quadratic and linear relationships with Inter Stimulus Intervals from CPT and Go/No Go tasks. Furthermore, tasks and cognitive domains influence the three parameters. Interpretations of ex-Gaussian parameters and clinical implications of these findings are also discussed. Fitting ex-Gaussian distributions to RT data is a useful way to explore differences between individuals with ADHD and healthy controls.

Keywords: ADHD; reaction times; ex-Gaussian; inter-trial variability.

1. Introduction

Attention Deficit/Hyperactivity Disorder (ADHD) is one of the most prevalent neurodevelopmental disorders in childhood and adolescence, with a prevalence ranging from 4 to 8% (Polanczyk et al., 2014). ADHD is characterized for three core symptoms: inattention, hyperactivity, and impulsivity (Barkley, 1997). Furthermore, patients with ADHD show impairments in several cognitive domains which impact daily activities. In the context of laboratory tasks, it has been consistently observed that individuals with ADHD show larger intra-individual variability in response times (RT) than healthy controls (Castellanos et al., 2006; Kofler et al., 2013; Levy et al., 2018; Tamm et al., 2012). This increased intra-individual variability has been proposed as a candidate endophenotype for ADHD (Castellanos et al., 2005; Henríquez-Henríquez et al., 2015; Karalunas et al., 2014; Lin et al., 2015; Salunkhe et al., 2020; 2021).

Means from RT distributions are often used in clinical research, but means alone do not account for the shape of the distribution (Dawson, 1988; Heathcote et al., 1991). Also, estimating means and standard deviations implicitly assumes that RT distributions are close to Normal distributions, but RT empirical distributions seldom resemble Normal curves; rather, these distributions are usually positively skewed (Luce, 1986; Van Zandt, 2000). Using the mean (or the median) as central tendency statistics alone may conduce to biases and increase the risk of falsely reject null hypotheses (Morís Fernández & Vadillo, 2020; Rousselet & Wilcox, 2020). Furthermore, the mean and the standard deviation of RT distributions are strongly correlated (Luce, 1986; Wagenmakers & Brown, 2007); if standard deviation, as a measure of intraindividual RT variability, is larger for people with ADHD, then this effect may also inflate the RT means for people with ADHD.

Among other alternative approaches (Ging-Jehli et al., 2021), it has been proposed using a theoretical distribution to describe and compare the shapes of different RT distributions (Castellanos et al., 2006; Van Zandt, 2000). The most widely used theoretical distribution in ADHD research is the ex-Gaussian distribution. The ex-Gaussian distribution is the convolution of the Gaussian and the exponential distributions. It is defined by three parameters: *µ, σ,* and *τ*. Parameters *µ* and *σ* derive from the Gaussian mean and standard deviation µ and σ are related to the faster, more regular responses. τ is related to the atypically slow responses. Figure 1 describes in more detail the effect of the parameter values on the shape of the ex-Gaussian distribution. Unlike mean and standard deviation, fitting ex-Gaussian distributions to empirical RTs allow to separate RT between a speed component, µ, and two variability components, σ and τ (Heathcote, 1996). These components are independent on each other, in contrast to RT mean and variance (Salunkhe et al., 2020). Furthermore, as pointed out below, σ and τ have different meanings. Nonetheless, fitting ex-Gaussian models to RT data is not always recommendable: first, a minimum of 100 observations is necessary to get reasonably reliable estimates (Heathcote, 1996; Lacouture & Cousineau, 2008; Van Zandt, 2000). Also, for sporadic participants, ex-Gaussian fit to RT data may be poor due to their proximity to Normal distribution (Lacouture & Cousineau, 2008; Yamashita et al., 2021).

The seminal work by Leth-Steensen et al. (2000) first used the ex-Gaussian distribution in order to perform what they called “response time distributional approach”, that is, to take into account the whole RT distributions (and not merely mean and standard deviation) by comparing the ex-Gaussian parameter values from ADHD patients and healthy controls. They found that children with ADHD presented larger τ values than controls, what they named “tau effect”. This effect was attributed to a greater proportion of slow reactions from children with ADHD compared to controls. Since then, many studies applied the ex-Gaussian distribution to account for the whole RT distributions when comparing patients with neurodevelopmental disorders and control groups. Although the “tau effect” is generally confirmed (Karalunas et al., 2014; Kofler et al., 2013; Salunkhe et al., 2020), it is less clear the effect of the two other parameters. Variables like age (Galloway-Long et al., 2021; van Belle, van Hulst, et al., 2015; van Belle, van Raalten, et al., 2015), gender (Patros et al., 2018; Seymour et al., 2016) or cognitive task or domain (Epstein et al., 2011; Ging-Jehli et al., 2021) have been hypothesized to modulate these differences in RTs.


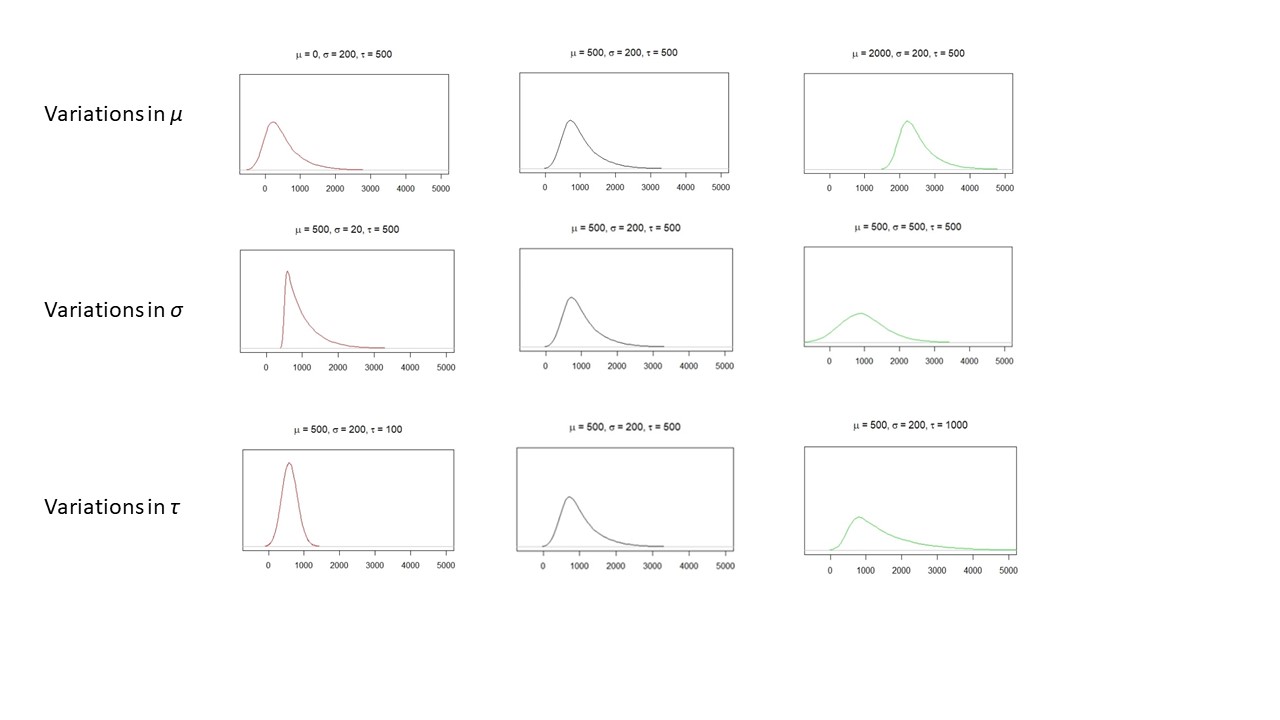


Figure 1: Shapes of ex-Gaussian distribution as parameter values are altered. The rows correspond to parameters µ, σ, and τ, respectively. From left to right, the parameter value goes larger. It can be seen that parameter µ mainly affects the position of the distribution. Parameter σ is mostly related to the width (dispersion) of the left part of the distribution; greater values for parameter σ lead to wider distribution. Parameter τ affects the height of the left part of the distribution and the thickness of the tail; larger values of τ lead to lower left parts and heavier tails. Graphs were made using “gamlss.dist” package for R (Stasinopoulos et al., 2021).

Although ex-Gaussian distribution is often used as a purely descriptive tool for empirical reaction time distributions (Fitousi, 2020; Matzke & Wagenmakers, 2009; Rieger & Miller, 2020), trying to find correlates of ex-Gaussian parameters to cognitive processes is tempting, as it would help to improve parameter interpretation and diagnosis through tasks using RT distributions. For instance, Yamashita et al. (2021) suggested that exGaussian distribution separated reaction times in a strategy factor (µ), a variability factor (σ), and a long-tail factor (τ).

In this sense, parameter τ has received the most attention, consistently with the greater relevance of τ in ADHD research. Several interpretations were proposed. The most usual interpretation for parameter τ is that longer τ reflects more frequent and pronounced attentional lapses during task performance (Leth-Steensen et al., 2000; Salunkhe et al., 2020). Anyway, other possible meanings for τ have been proposed. Based on state-regulation model (Sergeant, 2005), parameter τ may also be related to cognitive energy regulation impairments, which are hypothesized to be larger for people with ADHD . On other side, studies based on information accumulation models models suggest that τ parameter may be related to impaired speed of information accumulation in patients with ADHD (Fitousi, 2020; Matzke & Wagenmakers, 2009). Research on the relationship between ex-Gaussian parameters and race models partly support the relationship between τ and information accumulation rates. Parameter µ has a more straightfoward substantive interpretation. Variations in µ displace the whole RT distribution without altering its shape, thus relating µ with reaction speed. In general terms, lower µ values reflect faster responses and higher values correspond to slower responses. µ may be influenced by speed-accuracy trade-offs (Yamashita et al., 2021). Lower values for µ have been related to impulsive behavior in ADHD patients. Last, parameter σ has been hypothesized to be related to motor timing and/or impaired response preparation (Leth-Steensen et al., 2000), perhaps as a consequence of poor neuro-modulation (Salunkhe et al., 2020). Yamashita et al. (2021) found a moderate correlation between σ and both omission and comission errors, which led them to hypothesize a relationship between σ values and mind-wandering.

The aim of the present meta-analysis is to synthesize the available evidence on the comparative of ex-Gaussian distribution parameters estimated from RT data between people with ADHD and controls, examine the effect of ADHD on the whole RT distribution, and examine the influence of potentially moderator variables.

1. Methods
   1. Search strategy and syntaxes

We conducted searches on databases PubMed, PsycInfo, PsycArticles, Embase, Cochrane, Web of Science, and Scopus. Search syntaxes were similar to “ex-Gaussian” OR “exGaussian” (free language) AND “Attention Deficit/Hyperactivity Disorder” (term from thesaurus when applicable). We also searched for so-called “grey literature” in OpenGrey, OpenDOAR, WorldCat, and Google Scholar, in order to reduce risks of publication bias (Conn et al., 2003; McAuley et al., 2000; Siddaway et al., 2019). Searches were last performed on 7^th^ April 2022. This meta-analysis was registered in PROSPERO (CRD42021277339).

- 1. Inclusion and exclusion criteria

We included in the present meta-analysis studies with: a) Observational or quasi-experimental designs with at least one sample of patients diagnosed with ADHD and one control group; b) including estimations for at least one of the three ex-Gaussian parameters and contrast of differences between patients with ADHD and controls; and c) providing sufficient information to extract or estimate Cohen’s d as effect size. Not providing contrasts between ADHD group and control group or not providing sufficient data to estimate an effect size assimilable to Cohen’s d resulted in exclusion. Non-primary studies (e. g. reviews and meta-analyses) were also excluded. Last, for σ and τ estimations, only studies with at least 30 participants on each group were included (see “Data extraction” section below).

Two authors (MBF and MMM) independently selected studies through Rayyan platform. Disagreements were resolved reaching a consensus.

- 1. Data extraction

We analyzed differences in the ex-Gaussian parameters between groups of patients with ADHD and control groups. These differences are usually analyzed in the primary studies through t-tests or ANOVA tests, from where Cohen’s d can be calculated as effect sizes. Unstandardized effect size measures may seem appropriate for this meta-analysis since the ex-Gaussian parameters from RT distributions are usually on the same measure units (milliseconds or, less frequently, seconds). Nonetheless, magnitudes vary widely across tasks and samples: µ varies from 200 to almost 1000 ms, σ varies from 15 to 250 ms, and τ varies from 30 to 400 ms. This heterogeneity in scale measures makes more suitable using standardized effect size measures (Cheung & Vijayakumar, 2016). Positive values of d indicate a greater parameter value for the ADHD sample, and conversely, negative values of d indicate a greater parameter value for the control sample.

Using Cohen’s d as effects sizes requires sample distributions of the variables to be Normal. Being µ the mean of the Gaussian part of the distribution, it is clear that µ is theoretically Normal. On the contrary, σ and τ are not (Gmehlin et al., 2014; Leth-Steensen et al., 2000): sample means of σ and τ are Gamma distributed; in fact, σ fits a Chi-square distribution, a particular case of Gamma distributions. Normal distributions are a good approximation for Gamma distributions with sample sizes larger than 30 (Johnson & Kotz, 1970). For this reason, we included in analysis for σ and τ only those studies with sample sizes of 30 or more participants on each group.

For every study, if Cohen’s d were provided, they were directly taken. Otherwise, if mean and standard deviation (or analogous measurements like standard error for means) were provided, Cohen’s d was calculated through Psychometrica (Lenhard & Lenhard, 2016). Otherwise, if statistic tests and sample sizes were provided, Cohen’s d were calculated. Otherwise, if *η^2^* were provided, d were calculated following Cohen (1988); in these cases, the effect direction was determined observing which sample mean was larger: the effect was positive if the ADHD sample had a larger mean, and negative if the control group has a larger mean. If none of the above were provided, corresponding authors were contacted for further data.

Five studies (Duffy et al., 2021; Halliday et al., 2021; Keith et al., 2017; Thissen et al., 2014; Zhao et al., 2021) only reported analyses for τ. Four other studies (Borella et al., 2013; Gmehlin et al., 2016; Osmon et al., 2018; Rosch et al., 2013) reported non-significant effects for µ, without providing further data; In these cases, as recommended in Rosenthal (1995), they were assigned effect size values to 0, rather than excluding the study and thus incrementing the bias risk. Anyway, we performed a sensitivity analysis to study what would have happened with the estimation of µ effect (see Appendix 1). In summary, retaining these four studies has little effect on the estimation of µ effect or the risk of bias, as seen in Figure S1.

In every case, intra-study variances were calculated from Cohen’s d and sample sizes through the corresponding equation.

- 1. Data analysis

All the analyses were performed through Metafor package for R (Viechtbauer, 2010). First, we grouped outcomes in clusters according with the following rules: two outcomes from the same study belong to the same cluster, and outcomes from two different studies which share at least one author belong to the same cluster, unless the authors declared there was no overlapping between samples or we could reasonably assume the lack of overlapping between samples. We identified seven clusters of studies sharing one or more authors. In these cases, we contacted the authors to ask them regarding sample overlap. Three of them declared there was no between-study sample overlapping. Another cluster contained two studies with a large time lapse between them and we decided that there cannot be overlapping between their samples. The three remaining clusters were treated as with overlapping samples. See Appendix 2 for details. Once the clusters were established, three-level models were performed to obtain pooled effect size estimations, with the cluster as a random effect (Assink & Wibbelink, 2016; Van den Noortgate et al., 2015). Overall results were displayed in a forest plot. The same procedure was followed when analyzing subsets of studies/clusters defined by the cognitive domains. Also, three-level mixed-Effects models were fitted to explore the relationship between the effect sizes and a group of potentially moderator variables. Pseudo-R^2^ estimations (Cheung, 2015) were performed for each parameter and variance component through the following equations: $R_{W}^{2}=\frac{s_{W:M1}^{2}-s_{W:M2}^{2}}{s_{W:M1}^{2}}$ [1] and $R_{B}^{2}=\frac{s_{B:M1}^{2}-s_{B:M2}^{2}}{s_{B:M1}^{2}}$ [2], where R^2^_W_ and R^2^_B_ are the pseudo-R^2^ for within-cluster and between-cluster components, s^2^_W:M1_ and s^2^_B1_ are the within-cluster variance component of the model without moderator, and s^2^_W:M2_ and s^2^_B:M2_ are the within-cluster and between-cluster variance components of the model with moderator. Negative values for pseudo-R^2^ were set to 0

Heterogeneity between studies was assessed through the within-cluster and between-cluster components of variance. The significance of each variance component was determined performing log-likelihood-ratio tests comparing the variances of the full models with the variances of models whose within-cluster or between-cluster variances, respectively, were fixed to 0 (Assink & Wibbelink, 2016).

Regarding potential publication bias, regression tests, trim-and-fill methods, and estimation of fail-safe numbers are the most common methods for bias assessment. Nonetheless, when outcomes are not independent, some of these methods are discouraged. In this sense, following recommendations from Nakagawa et al. (2022), we performed funnel plots and tested their significance through multi-level meta-regression models using sample size as a fixed-effect moderator variable. Using sample sizes seems to outperform using standard errors with non-independent outcomes (Fernández-Castilla et al., 2021; Nakagawa et al., 2022). More specifically, these authors propose to use the inverse of the “effective sample size” instead of the sum of the sample sizes. The effective sample size is calculated as follows: $ñ_{i}=\frac{n_{1i}n_{2i}}{n_{1i}+n_{2i}}$, where ñ_i_ is the effective set size of study I, n_1i_ is the sample size of the group 1 in study I, and n_2i_ is the sample size of the group 2 in study i. We first used the square root of the inverse of the effective set size, $\sqrt{\frac{1}{ñ_{i}}}=\sqrt{\frac{n_{1i}+n_{2i}}{n_{1i}n_{2i}}}=\sqrt{\frac{1}{n_{1i}}+\frac{1}{n_{2i}}}$ , to test the significance of funnel asymmetries.

1. Results
   1. Overall results

A total of 51 studies were included in the present meta-analysis. The flow diagram is presented in Figure 2.


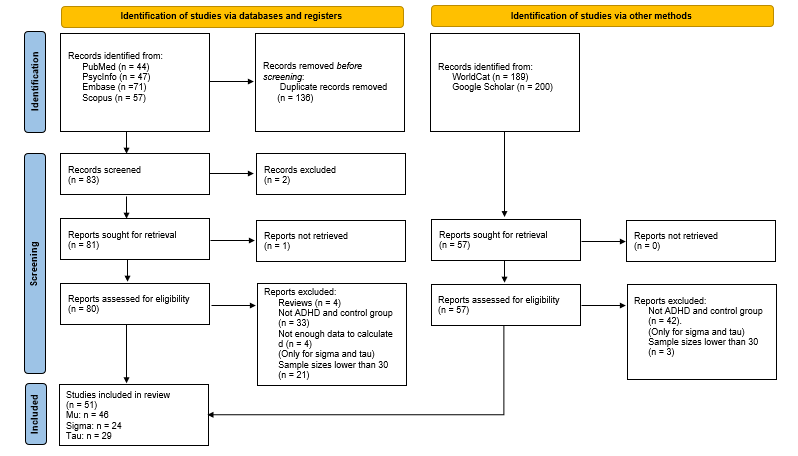


Figure 2: Flow diagram of searches for this meta-analysis.

The overall results for this meta-analysis are summarized in the forest plots in Figure 3 (µ), Figure 4 (σ), and Figure 5 (τ). The pooled effect for *µ* was *d*=0.0447 (95% CI: [-0.0399,0.1292]). Thus, our meta-analysis found no general differences in *µ* value between ADHD and control groups. These general differences were found to be moderate for *σ* (*d*=0.2551, 95% CI: [0.1441,0.3660]) and moderate-to-large for *τ* (*d*=0.5294, 95% CI: [0.3992,0.6597]), according to an usual interpretation for Cohen’s d (Cohen, 1988). Heterogeneity was estimated to be statistically significant for all parameters and variance components, except for the between-cluster variance of mu (Table 1), which confirmed the convenience of estimating a random effects model for the three parameters, as well as indicating that a number of variables may be moderating the differences for the three ex-Gaussian parameters.

Table 1. Within-cluster and between-cluster heterogeneity estimations for the ex-Gaussian parameters.

|  | Within-cluster variance | Significance | Between-cluster variance | Significance |
| --- | --- | --- | --- | --- |
| *µ* | 0.0359 | <.0001 | 0.0182 | .0574 |
| *σ* | 0.0077 | .0346 | 0.0247 | <.0001 |
| *τ* | 0.0246 | <.0001 | 0.0447 | <.0001 |


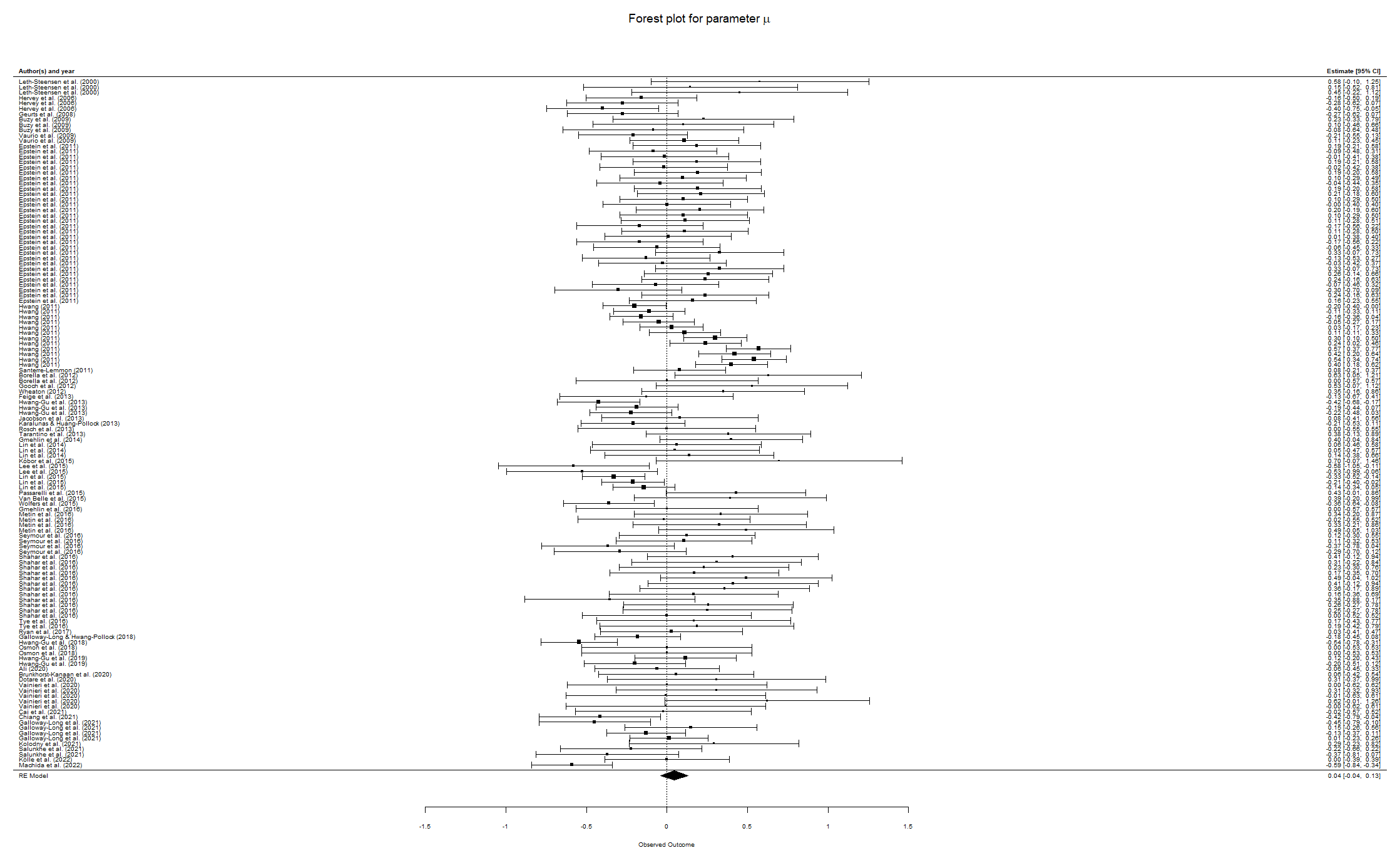


Figure 3: Forest plots for parameter µ. Parameter µ shows no global differences between ADHD patients and healthy controls.


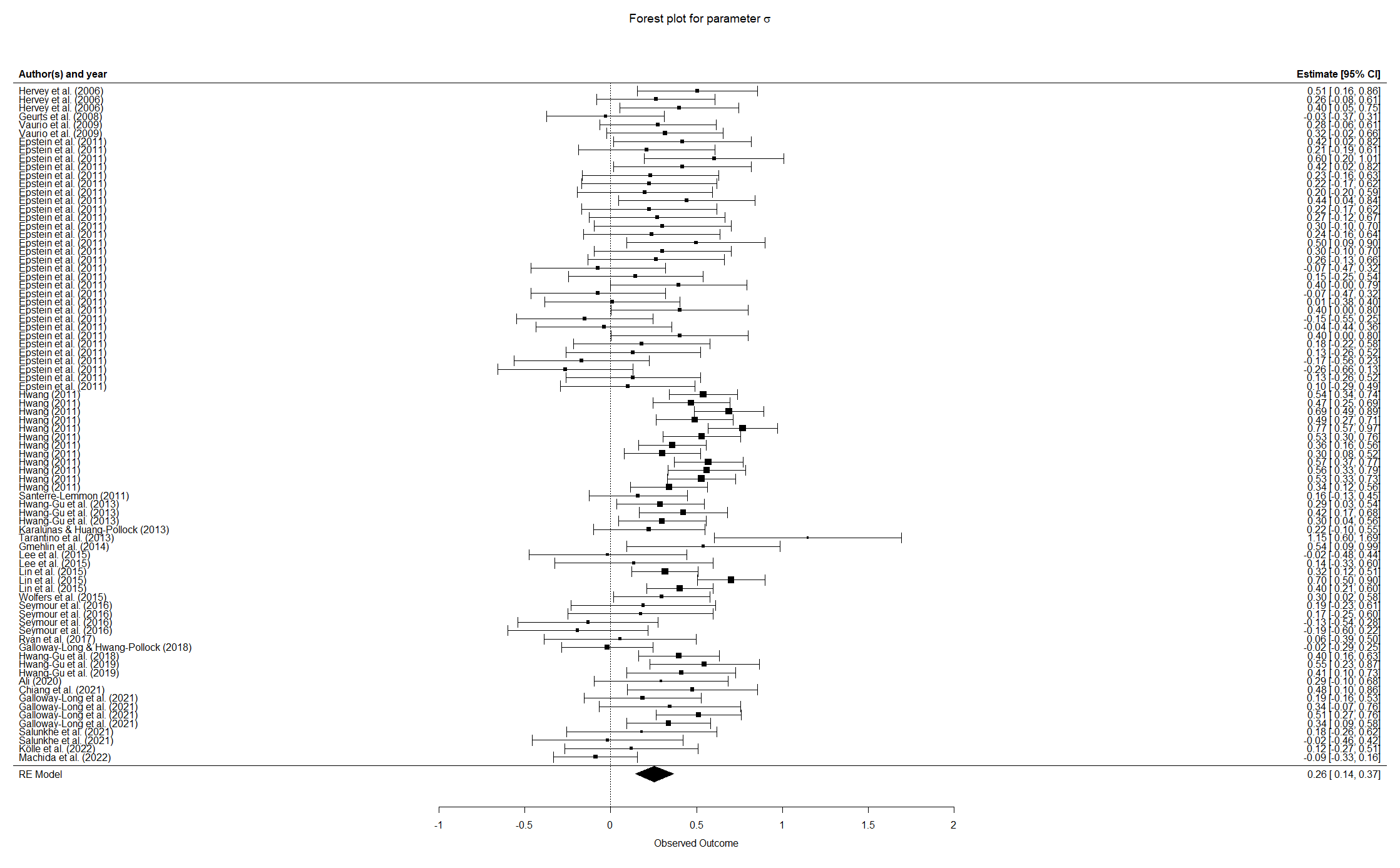


Figure 4: Forest plot for parameter σ.


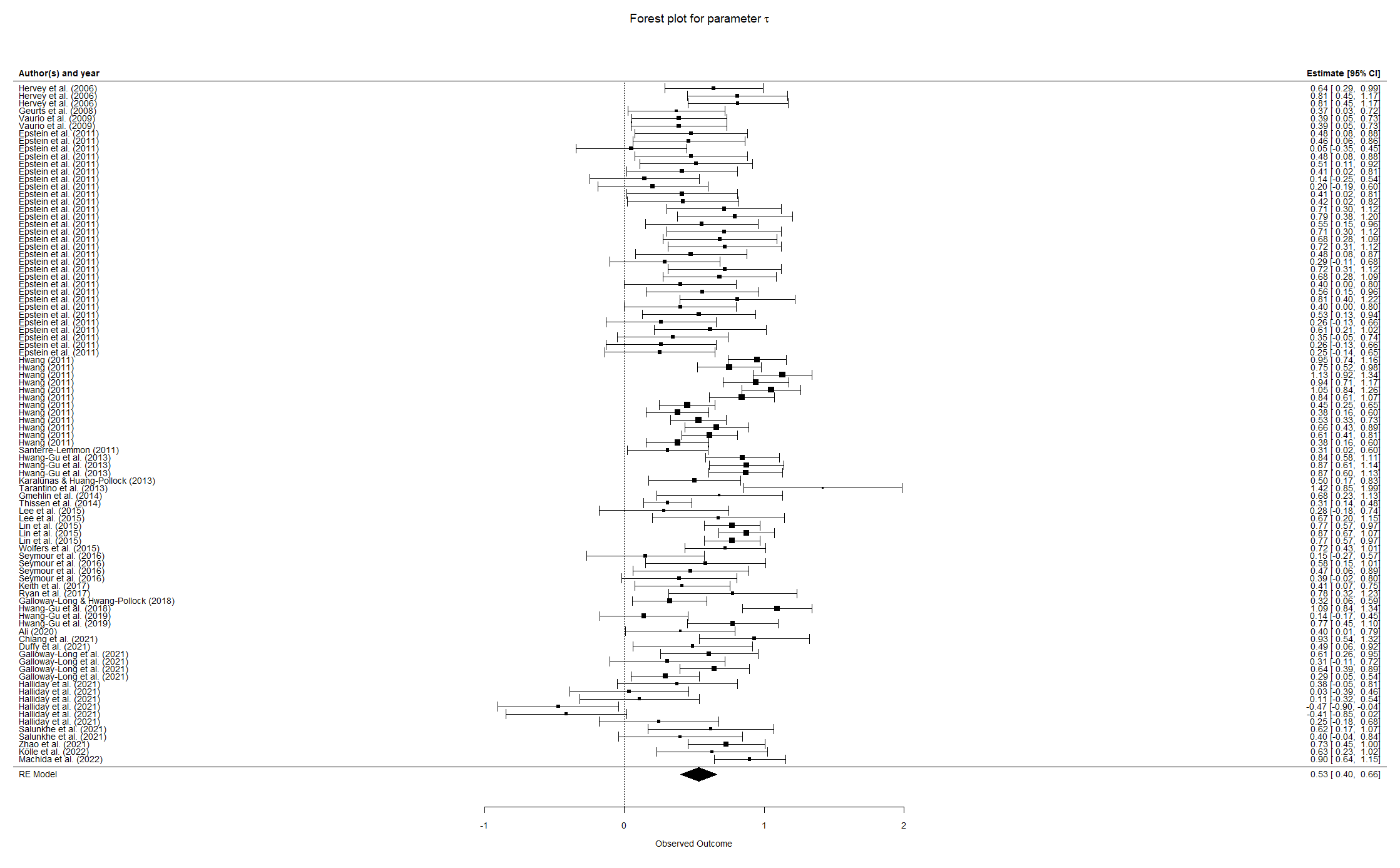


Figure 5: Forest plot for parameter τ.

- 1. Analysis of moderator variables
     1. ADHD subtype

Twenty of the included studies differentiated between three ADHD subtypes: predominantly Hyperactive, predominantly Inattentive, and mixed or combined. These three subtypes differ in terms of their symptomatology and thus may present differences in cognitive tasks. Only one of these studies (Epstein et al., 2011) explicitly used the ADHD subtype as a factor to explore differences in ex-Gaussian parameters, but the rest of them differed in the reported proportion of patients with each ADHD subtype. We coded these reported proportions and performed a meta-regression analysis. Of course, for every primary study, the proportions of patients for each ADHD subtype are not independent variables; rather, one of the proportions is a linear combination of the two others. Thus, we estimated meta-regression models for the three parameters taking the proportion of patients with Hyperactive ADHD subtype and the proportion of patients with Inattentive ADHD subtype as moderator variables.

Meta-regression models did not account for a significant proportion of variability for µ (QM(2) = 0.8802, p = .6440, R^2^_W_ = 0.2880, R^2^_B_ = 0). For σ the meta-regression was significant (QM(2) = 7.8783, p = .0195, , R^2^_W_ = 0.6472, R^2^_B_ = 0) and the meta-regression coefficient for the proportion of Inattentive subtype was significant (β = -0.1397, p = .0064). For τ the meta-regression was not significant (QM(2) = 3.3368, p = .1885, , R^2^_W_ = 0, R^2^_B_ = 0), but the coefficient for inattentive subtype approached significance (β = -0.1307, p = .0679). The meta-regression coefficients suggest that, the larger the proportion of inattentive ADHD patients in the clinical sample, the shorter the differences in parameter σ (and perhaps τ) between clinical and typical samples.

- - 1. Task / Cognitive domains assessed

Tasks were then classified for cognitive domains according to the classification used in Ging-Jehli et al. (2021), which contemplates six domains for the most common cognitive tasks: Cognitive flexibility, Selective attention, Working memory, Time perception, Sustained attention, and Inhibitory control. Every task was assigned to one or more cognitive domains. As observed in Castellanos et al. (2006), a predominance of “hot” executive functions, rather than “cool” functions, is also observed in the selected studies. Table 2 summarizes how we classified all the tasks used in the primary studies.

Table 2: Classification of tasks used in the included studies by cognitive domain (Ging-Jehli et al., 2021). It is noteworthy that some cognitive domains outnumber the total reviewed studies because several articles provided results for more than one experimental condition.

| Cognitive domain | Tasks included | Outcomes for every cognitive domain | | |
| --- | --- | --- | --- | --- |
|  |  | µ | σ | τ |
| Cognitive flexibility | Posner task  Flanker tasks  Stroop tasks  Change task | 9 | 1 | 1 |
| Selective Attention | Attentional Network task  Flanker tasks  Stroop tasks  Posner task | 15 | 6 | 6 |
| Working memory | n-back task  Classification tasks  Visual Serial Addition Task | 24 | 8 | 8 |
| Time Perception | Motor Timing task | 6 | 6 | 7 |
| Sustained attention | Simple reaction time tasks  Warned reaction time task  Fast task  Go task  Go/No go  Oddball task  Sustained Attention to Response task  WAF – Vienna Test Systems  Rapid Automatized Naming task  Choice tasks  Finger tapping task | 73 | 49 | 54 |
| Inhibitory control | Go/No go  Stop signal  Change task  Visual inspection time task  Stroop tasks  Continuous Performance Tests  Jelly bean task  Multi-source interference task | 66 | 51 | 59 |

We then generated six new dichotomic variables, one for each cognitive domain. Thus, for every study and task these variables account for whether or not the domains are measured. After aggregating outcomes by clusters, random effect models were estimated for each cognitive domain separately, in order to obtain effect size estimations for the differences between ADHD patients and healthy controls for every cognitive domain. These estimations are provided in Table 3.

For µ, only effect sizes for Selective Attention and Time Perception tasks were non-zero. For tasks involving Selective Attention, people with ADHD tended to show larger µ values. Also, differences (non-overlapping CI for effect sizes) were observed between Time Perception and both Selective Attention and Working Memory. In tasks involving Selective Attention and Time Perception, participants with ADHD tended to respond slower than controls.

For σ, estimated effect sizes for all cognitive domains except Working Memory and Cognitive Flexibility were significantly positive, which means that σ parameter tended to be larger for ADHD patients than for controls. These differences were larger for Time Perception and Selective Attention, and shorter for Inhibitory Control, Sustained Attention, and Working Memory.

For τ, estimated effect sizes for all cognitive domains were positive. As expected, τ parameter also tended to be larger for people with ADHD than controls. The τ effect is slightly variable across cognitive domains, being Sustained Attention and Inhibitory Control the domains where the effects were larger, and Cognitive Flexibility the domain where the effect was shorter.

Table 3: Estimated effect sizes and CI (95%) for µ, σ, and τ for cognitive domains. Significant differences are in bold characters.

|  | Cognitive Flexibility | Inhibitory Control | Sustained Attention | Selective Attention | Working Memory | Time Perception |
| --- | --- | --- | --- | --- | --- | --- |
| µ | 0.19  [-0.05, 0.43] | 0.01  [-0.13, 0.14] | -0.02  [-0.13, 0.10] | **0.20**  [0.08, 0.33] | 0.05  [-0.11, 0.20] | **0.42**  [0.31, 0.52] |
| σ | -0.03^*^  [-0.37, 0.31] | **0.27**  [0.14, 0.41] | **0.31**  [0.17, 0.44] | **0.23**  [0.07, 0.39] | 0.15  [-0.03, 0.33] | **0.45** [0.35,0.54] |
| τ | **0.37**^*^  [0.03, 0.72] | **0.53**  [0.36, 0.60] | **0.62**  [0.49, 0.76] | **0.49**  [0.33, 0.66] | **0.46**  [0.33, 0.60] | **0.42**  [0.24, 0.61] |

^*^The estimation is based on only one outcome.

- - 1. Age

For every study, mean age of ADHD and control group was recorded. We calculated the weighted mean of ages from the two groups to obtain the total sample mean age.

We fitted a mixed-effects model for each ex-Gaussian parameter with the sample mean age as a moderator variable. Regression models were not significant for µ (QM(1) = 0.5429, p = .4612, R^2^_W_ = 0, R^2^_B_ = 0.1648), σ (QM(1) = 0.0127, p = .9102, R^2^_W_ = 0, R^2^_B_ = 0) or τ (QM(1)=2.2164, p = .1365, R^2^_W_ = 0.0304, R^2^_B_ = 0). Differences in ex-Gaussian parameters between people with and without ADHD seem to keep approximately constant across ages.

- - 1. Gender

47 studies reported the proportions of males and females in the experimental groups. The vast majority of studies reported larger proportion of females in control groups than ADHD groups. Proportion of females in ADHD and control groups were found to be highly correlated (Spearman’s ρ = .870, p < .001) and thus the two moderators combined were not suitable to meta-regression. Thus, we fitted meta-regression models using only the proportion of females in ADHD groups. For these meta-regression models, positive values indicate larger female proportions.

The effect of gender proportion was not significant for µ (QM(1) = 0.4499, p = .5024, R^2^_W_ = 0.0026, R^2^_B_ = 0.0482), σ (QM(1) = 0.2640, p =.6074, R^2^_W_ = 0, R^2^_B_ = 0) or τ (QM(1) = 0.7971, p = .3720, R^2^_W_ = 0, R^2^_B_ = 0).

Also, the difference between female proportions was calculated for each study, as follows: $\Delta\left( \% Female \right)={\% Female}_{Control}- {\% Female}_{ADHD}$; this difference was used as a moderator variable for meta-regression models. Positive values indicate a larger female proportion in Control group. For µ and σ, the meta-regression model with the proportion difference as a predictor variable accounted for a non-significant amount of variability and their respective coefficients were not significant for σ (QM(1) = 2.4014, p = .1212). The regression coefficients were significant for µ (QM(1) = 9.1929, β = 0.0094, p = .0024) and τ (QM(1) = 5.9540, β = -0.0083, p = .0147).

- - 1. Interstimuli interval (ISI)

In 21 studies, mostly using Go/No Go and CPT tasks, interstimuli intervals (ISI) are reported. We estimated meta-regression models with the ISI as a predictor variable. The coefficient of linear regression approaches significance for σ (β = -0.0392, p = .0505, R^2^_W_ = 0, R^2^_B_ = 0), but is not significant for µ (p = .0970, R^2^_W_ = 0.9421, R^2^_B_ = 0) or τ (p = .2965, R^2^_W_ = 0.5823, R^2^_B_ = 0.2574).

We also estimated quadratic meta-regression models for the three parameters. For none of them the non-intercept coefficients of µ and σ were statistically significant (all ps > .1 except the second-grade coefficient for sigma: β_2_ = -0.0293, p = .0607), but the coefficients associated with the quadratic model on τ are statistically significant (β_1_ = -0.2655, p = .0042; β_2_ = -0.0428, p = .0075). Figure 6 shows the meta-regression lines.


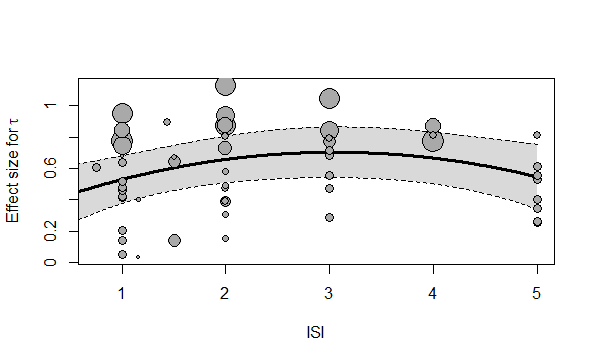


Figure 6: Meta-regression curve for τ. The predictor variable is ISI. The quadratic relationship between ISI and tau somehow resembles the one predicted by the State Regulation hypothesis (Metin et al., 2016)

These results suggest that ISI does not alter the difference on the usual reaction speed, but on intertrial variability. On one side, as ISI increases, differences in intertrial variability of usual responses tend to mitigate; on the other side, there seems to be an optimum of differences in reaction time lapses at around 3 seconds of ISI.

- - 1. Intelligence Quotient (IQ)

A total of 35 studies reported sample IQs and 28 studies of them excluded participants with IQ below a certain threshold. We compared the estimations of studies with and without this IQ-based exclusion criterion. For none of the parameters the existence of an IQ threshold supposed a significant difference (µ: QM(1) = 0.1423, p = .7060, R^2^_W_ = 0.0595, R^2^_B_ = 1; sigma: QM(1) = 0.1328, p = .7156, R^2^_W_ = 0.0283, R^2^_B_ = 0; tau: QM(1) = 0.0001, p = 0.9917, R^2^_W_ = 0, R^2^_B_ = 0.4711). However, among those studies which did applied an IQ threshold, there was a significant effect of the IQ threshold value on the effect of µ (QM(1) = 12.8246, β = 0.0319, p = .0003, R^2^_W_ = 0.0085, R^2^_B_ = 0), but not σ (QM(1) = 1.5757, p = .2094, R^2^_W_ = 0.0120, R^2^_B_ = 0) or τ (QM(1) = 0.0361, p = .8492, R^2^_W_ = 0, R^2^_B_ = 0). Figure 7 shows the meta-regression for µ and how a threshold of 80 seems to separate positive and negative differences in µ.

We also analyzed a possible effect of the difference in IQ between participants with and without ADHD. No significant results were obtained for sigma or tau (sigma: QM(1) = 0.1629, p = 0.6865, R^2^_W_ = 0, R^2^_B_ = 0.1105; tau: QM(1) = 0.4766, p = 0.4900, R^2^_W_ = 0.5746, R^2^_B_ = 0.3230), but mu showed a negative significant effect (QM(1) = 4.8373, β = -0.1996, p = .0279, R^2^_W_ = 0.9886, R^2^_B_ = 0), as described in Figure 7.


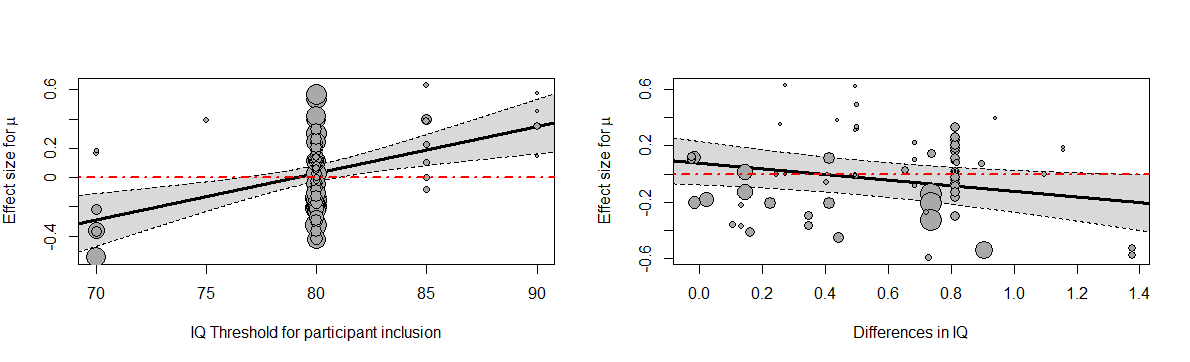


Figure 7: Meta-regression plots for µ as a function of IQ threshold of exclusion criterion and differences in IQ between samples, respective. An IQ threshold of 80 is the most widely used. Thresholds below 80 tend to result in faster reactions from patients with ADHD and thresholds above 80 tend to result in faster responses from people without ADHD. Regarding differences in IQ, only differences below 0.4 IQ points are related with slower reactions from people with ADHD, while, as these differences become larger, the groups of patients with ADHD tend to respond faster than groups of people without ADHD.

- - 1. Stimulant medication use

Regarding medication use, 39 studies asked those participants with ADHD who were being treated with stimulants to discontinue this treatment 24 or 48 hours prior to participate in the studies. Other 5 studies directly excluded those participants with ADHD who were taking stimulants. The rest of the studies did not apply any rule regarding medication use. It is possible that these different manners to deal with medication among the samples has an effect on the estimations. To explore this possibility, we compared the estimations of these three study subgroups, using the medication modality as a moderator variable. We did not obtain significant differences in µ (QM(2) = 2.8482, p = .2407, R^2^_W_ = 0, R^2^_B_ = 0.1974), σ (QM(2) = 3.2372, p = .1982, R^2^_W_ = 0, R^2^_B_ = 0) or τ (QM(2) = 4.1879, p = 0.1232, R^2^_W_ = 0.0562, R^2^_B_ = 0).

- - 1. Comorbidities

A large number of comorbidity-related exclusion criteria were used in one or more of the included studies. Among those exclusion criteria, we selected the more frequent and/or more commonly related with ADHD to test whether adding these exclusion criteria could affect the differences on parameters. These results are summarized in Table 4.

Table 4: Moderator analysis using the presence or absence of comorbidities as exclusion criteria.

|  | Neurological disorders | | Mood disorders | | Anxiety disorders | | Autism Spectrum Disorders | | Schizophrenia or Psychosis | | Sensory or motor impairments | | Obsessive Compulsive Disorder | |
| --- | --- | --- | --- | --- | --- | --- | --- | --- | --- | --- | --- | --- | --- | --- |
|  | QM(1) | P value | QM(1) | P value | QM(1) | P value | QM(1) | P value | QM(1) | P value | QM(1) | P value | QM(1) | P value |
| µ | **7.5223** | **0.0061** | 0.7786 | 0.3776 | 2.3781 | 0.1230 | **8.6614** | **0.0033** | 0.8338 | 0.3612 | 1.0638 | 0.3024 | 0.0317 | 0.8588 |
| σ | 3.0585 | 0.6079 | 0.7090 | 0.3998 | 0.6507 | 0.4199 | 0.3752 | 0.5402 | **5.1457** | **0.0233** | 2.2906 | 0.1302 | 0.8652 | 0.3523 |
| τ | 0.0165 | 0.8978 | 0.4191 | 0.5174 | 0.6519 | 0.4194 | 0.8338 | 0.3612 | 3.2620 | 0.0709 | 0.4414 | 0.5064 | 0.4286 | 0.5127 |

Only excluding patients with schizophrenia or psychosis had a statistically significant impact on σ estimation. The σ effect estimated on studies excluding patients with schizophrenia or psychosis was 0.4058 (95% CI: [0.2923, 0.5192] and 0.2120 (95%CI: [0.0752, 0.3489]) on studies not excluding them. Excluding patients with schizophrenia or psychosis reduced the variability in usual reactions. Also, excluding patients with neurological disorders or autism spectrum disorders (ASD) had a significant impact on mu estimations. The µ effect estimated on studies excluding patients with neurological disorders was 0.0575 (95% CI: [-0.0477, 0.1626] and -0.0230 (95%CI: [-0.1600, 0.1140]) on studies not excluding them. In turn, the µ effect estimated on studies excluding patients with ASD was -0.0578 (95% CI: [-0.2085, 0.0929] and 0.0556 (95%CI: [-0.0400, 0.1512]) on studies not excluding them. Excluding patients with neurological disorders and with ASD seem to have opposite effects: excluding patients with neurological disorders makes the ADHD group faster than the control group, while excluding patients with ASD makes the ADHD group slower than the control group.

- 1. Risk of bias

As described in the Methods section, publication bias was assessed through funnel plots and meta-regression models using effective sample size as a moderator variable.

Funnel plot asymmetry was statistically significant for µ (QM(1) = 10.4859, p = .0012) and was statistically non-significant for σ (QM(1) = 0.8617, p = .3533) and τ (QM(1) = 0.5104, p = .4750). Parameter τ received the most attention in ADHD research, to the point that 10 out of 139 outcomes (7.19%) only reported estimations for τ. Most likely, publication bias should be noticeable in τ parameter.

Funnel plots in Figure 8 show a small-study effect for µ, in the sense that effects tend to be positive for smaller studies, approximately zero for medium-sized studies, and negative for larger studies. Only larger studies tend to show statistically significant results, which points against a publication bias mediated for significance.


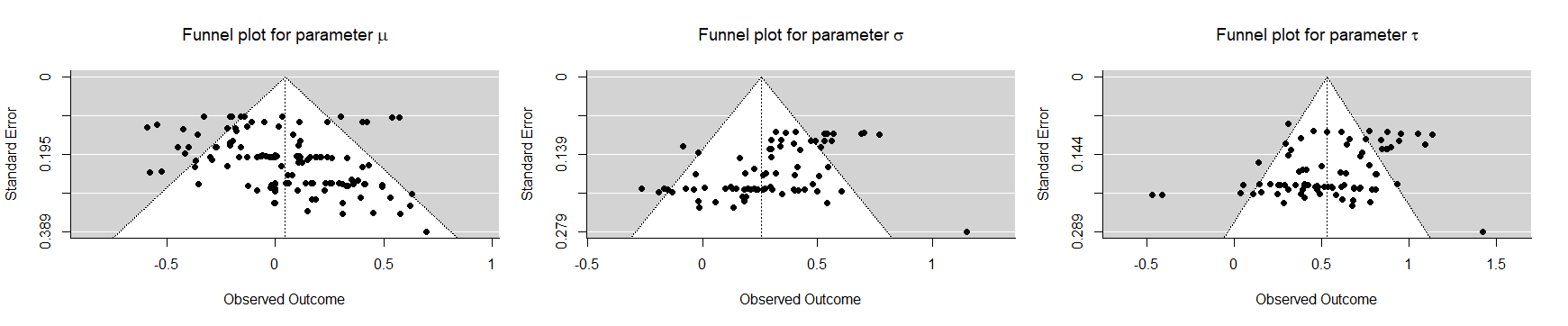


Figure 8: Funnel plots for asymmetry and risk of bias.

Clearly, meta-analysis for parameters σ and τ is not biased. On the contrary, estimation for parameter µ is strongly biased to the right. In this case, an overall estimate can be obtained through a meta-regression model with the inverse of the effective sample size, $\frac{1}{ñ_{i}}$, as a fixed effect. In our case, this regression model for mu gave an overall estimation of -0.1724 [-0.3014,-0.0434].

- 1. RT means and standard deviations

Last, we analyzed the differences on RT means and standard deviations between people with and without ADHD from those studies which gave these data. The effect size of the differences in RT mean was 0.4213 (95% CI: [0.2759,0.5668]), while for RT standard deviations the difference was 0.6025 (95% CI: [0.4886,0.7164]). These results suggest that patients with ADHD tend to have RTs with larger means and deviations. Also, both RT mean and standard deviation may discriminate between people with and without ADHD.

1. Discussion

Overall, we can conclude that the “τ effect”, as named by Leth-Steensen et al., (2000), as well as its homologous “σ effect”, are generally confirmed. τ effect is greater than σ effect. Studies combined show a lack of evidence for a “µ effect”, but this finding is biased in some extent. RT analysis based on ex-Gaussian parameters has been proposed for classification purposes to discriminate between individuals with and without ADHD (Galloway-Long et al., 2021; Hernaiz-Guijarro et al., 2019; Leth-Steensen et al., 2000; Wheaton, 2012), but a lack of discrimination power has also been reported (Brunkhorst-Kanaan et al., 2020). Our findings are consistent with the usage of either intertrial variability parameters, σ or τ for screening purposes. Our results also suggest that parameter µ -the central tendency of regular responses- is generally not discriminative.

In general, high levels of σ and τ may indicate the presence of ADHD symptomatology. This is consistent with the usage of intertrial variability for detecting ADHD and argues against the use of usual descriptive statistics (i. e., mean and standard deviation). Also, differences in RT means and standard deviations have the same direction (larger for patients with ADHD) and similar effect sizes. These results would suggest that RT of patients with ADHD are larger and more variable. Nonetheless, our analysis with ex-Gaussian parameters only suggest larger inter-trial variability, without clear differences in speed. In fact, regarding µ, although our meta-analysis shows a general lack of difference between patients with ADHD and controls, unbiased results point towards a slight general µ effect in the inverse direction than the two other effects, it is, ADHD samples tend to show lower values for µ.

- 1. Cognitive domains

Our analysis for cognitive domains revealed that tasks involving Selective Attention and Time Perception show effects on the three ex-Gaussian parameters. Impaired selective attention or, more specifically, increased distractibility, is a characteristic of patients with ADHD (Ging-Jehli et al., 2021). Our results indicate that patients with ADHD tend to be slower than people without ADHD in these tasks and suggest using RTs in these kind of tasks to discriminate between people with and without ADHD, but do not themselves determine the origin of this increased reaction time. Particularly noteworthy are the results on Time Perception, which show moderate effects on the three parameters. Two recent reviews (Nejati & Yazdani, 2020; Ptacek et al., 2019) show that impairments in time perception on patients with ADHD are significant and present, although with varying intensity, across time-related task types (time discrimination or estimation) and sensorial modalities. Ptacek et al. (2019) even propose time perception impairment as a diagnostic criterion for ADHD. Our results are consistent with this proposal. Sustained Attention and, in a less clear manner, Inhibitory Control may also serve to discriminate between people with and without ADHD, but only taking into account the inter-trial RT variability. On the other hand, tasks involving Working Memory and Cognitive Flexibility only show effect for τ. It is noteworthy that only one outcome was included in the analysis of sigma and tau effect on Cognitive Flexibility. Further research is needed to better establish the characteristics of RT distributions of people with ADHD in tasks involving cognitive flexibility. Although evidence finds impairments in working memory in patients with ADHD (Alderson et al., 2013; Kasper et al., 2012), our results only supports its detection through increased tau.

- 1. Age and Gender

Our results suggest that differences in intra-individual variability (σ and τ) are significant between ADHD and control groups, and these differences seem to keep constant across developmental periods. This result is consistent with the previous finding that intra-individual variability in people with ADHD seem to be more similar to younger controls than to age-matched controls (Leth-Steensen et al., 2000; van Belle, van Hulst, et al., 2015), showing a possible developmental impairment in inter-trial variability in ADHD samples. Consistently, Castellanos et al. (2006) found that patients with ADHD have a less mature cerebral cortex. Also, Van Belle, van Raalten, et al. (2015) showed that intra-individual variability in people with and without ADHD is mediated by different brain regions with different developmental phases, which could explain the impairment in intra-individual improvements.

Our results also suggest that differences in µ and σ remain constant regardless the gender proportions or the differences in the gender proportions between ADHD and control groups. On the contrary, differences in τ become larger as the difference in the proportions of females decrease (and the differences in the proportions of male increase). Conversely, for parameter µ the effect is the opposite: the larger the differences in male proportions, the larger the effect of µ. Several reviews found evidence of greater impulsivity (Gershon, 2002; Hasson & Fine, 2012), hyperactivity (Gaub & Carlson, 1997; Gershon, 2002), and inattention (Gaub & Carlson, 1997; Gershon, 2002) among male patients than female patients. Hasson & Fine (2012) found that the difference between males with and without ADHD in CPT impulsivity measures was larger than the difference between females with and without. Taken together, this evidence supports the interpretation of parameters µ and τ in terms of impulsivity and/or inattention.

- 1. ISI

ISI was hypothesized to influence the differences in reaction times between patients with ADHD and controls (Hwang-Gu, Chen, et al., 2019; Hwang-Gu et al., 2013; Lee et al., 2015; Leth-Steensen et al., 2000; Metin et al., 2016). In particular, Hwang-Gu et al. (2019; 2013), based on results from Leth-Steensen et al. (2000), hypothesized a direct relationship between τ and ISI, as an effect of increasing cognitive load with increased ISI. Metin et al. (2016) hypothesized a quadratic (U-shaped) relationship between σ and ISI. Both linear and quadratic coefficients in regression with sigma approached significance, which could be taken as some sort of evidence supporting a relationship between sigma and ISI, in the line hypothesized (Metin et al., 2016). This quadratic relationship is more evident with tau. The sample sizes used in some of these studies may have contributed to a premature confirmation of the hypotheses, in particular those referred to σ and tau. It is noteworthy that the differential effect of ISI in reaction time variability is reduced when the ISIs are randomized (“jittered”) in a certain task (Lee et al., 2015; Ryan et al., 2010). It is possible that jittering ISIs reduces boredom in ADHD patients. Also, it is possible that predictable ISIs allow people with ADHD some sort of planning on inattentional lapses.

- 1. IQ

In general, our results do not find any relationship between IQ and differences in intertrial RT variability. We did find that, although imposing a lower threshold in participants IQ do not significantly affect the results, researchers should be careful if they do. Taking into account only studies with a lower threshold for IQ, we found that it may influence differences in usual reaction times: starting from 80 IQ points, the upper the threshold, the larger the differences between people with and without ADHD. Interestingly, including patients with IQ below 80 make the differences in mu to be negative, it is, groups of patients with ADHD tend to respond faster than groups of patients without ADHD.

The difference in IQ may also have an effect on differences in mu. Our results suggest that when samples have similar IQ people with ADHD tend to respond slower than people without ADHD and, when these differences are larger, people with ADHD tend to respond slower than people without ADHD. In the context of intelligence and RT, it is generally accepted that people with larger IQ tend to respond faster (Der & Deary, 2003; Matthews & Dorn, 1989; Ratcliff et al., 2008). Also, in almost all studies included in this meta-analysis, samples of participants without ADHD have larger mean IQ than samples of patients with ADHD. With all this, we should expect that larger differences in IQ lead to larger, positive differences in mu, contrary to what we found.

- 1. Medication and comorbidities

Our results show that the medication status have little impact on the differential performance of people with and without ADHD. This may indicate that people participating in this kind of experiments are generally following large-term therapies and withdrawing medication has little effect in performance on cognitive tasks. Also, we only found effects of excluding patients with schizophrenia, neurological disorders, and ASD. Excluding patients with schizophrenia or psychosis shortened the difference in intra-individual RT variability, consistently with literature suggesting a relationship between schizophrenia and increased intra-individual RT variability (Kaiser et al., 2008; Karantinos et al., 2014; Smyrnis et al., 2009; Vinogradov et al., 1998). When excluding participants with ASD, groups with ADHD tended to be faster than groups without ADHD, which suggested that participants with ASD are slower than control groups. Consistently, Salunkhe et al. (2021) observed that participants with both ADHD and ASD has larger mu than participants with only ADHD and that controls. However, the same authors found a similar effect with sigma and tau, which is not consistent with the lack of effect that excluding patients with ASD had in the present meta-analysis. Furthermore, Karalunas et al. (2014) reported increased inter-trial variabilities in RT only when patients with ASD also had ADHD. Last, excluding patients with neurological disorders resulted in negative differences in parameter mu, which may suggest that patients with neurological disorders respond faster, perhaps due to an increased impulsivity.

- 1. Cognitive interpretations for ex-Gaussian parameters

As pointed out in the introduction, τ is the most analyzed parameter in terms of interpretation. It is generally accepted that larger τ values denote larger and more frequent RTs in the tail of the distribution. This has been interpreted in terms of attentional lapses, impairments in cognitive energy regulation or in information accumulation speed. Shahar et al. (2016) state that attentional lapses, or “mind-wandering”, should be more or less constant across tasks; according to our results, differences in τ are similar across cognitive domains, in keeping with that interpretation for τ. Nonetheless, our analysis on ADHD subtypes suggested that τ is inversely related with inattention.

Our results suggest that σ differences are estable across age, gender, ISI, and ADHD subtypes. Only cognitive domains had any influence on the intensity of differences in sigma, being Time Perception and Selective Attention the domains where this difference is larger. This suggests that σ may be related with attentional control and rather than vigilance, cognitive change or memory processes. Yamashita et al. (2021) reported correlations between σ and errors in general (commision, omission, mind-wandering measures…), but the Principal Component Analysis performed by Salunkhe et al. (2020) show that σ and errors saturate in different components; these same authors argued elsewhere (Salunkhe et al., 2019) that σ may be related with neuromodulation or neural “noise”.

Mu is related with the usual reaction speed, which is influenced by impulsivity. Since impulsivity is a core symptom in ADHD, relating parameter µ with impulsivity is consistent with our finding that µ, and thus usual reaction times, tend to be faster for ADHD groups.

- 1. Clinical implications

Regardless the cognitive process, τ tends to discriminate between patients with and without ADHD. σ is less useful as a discriminative parameter. Regarding µ, in tasks involving sustained attention, people with ADHD tend to respond slightly faster than controls, while in tasks involving selective attention people with ADHD tend to respond slower. Overall, sustained attention seems to be the only cognitive process where the three parameters show differences between people with and without ADHD. Several recent studies (Brunkhorst-Kanaan et al., 2020; Galloway-Long et al., 2021; Machida et al., 2022) directly assessed the discriminative power of the three parameters through ROC curves. τ and σ were slightly discriminative, while discriminative power for µ was more irregular, ranging from modearte to non-discriminative values.

- 1. Strengths and Limitations

Some reviews and meta-analyses have been previously performed on RT comparisons between groups with and without ADHD (Ging-Jehli et al., 2021; Karalunas et al., 2014; Kofler et al., 2013; Salunkhe et al., 2020; Tamm et al., 2012). The present meta-analysis improves previous work in two ways: first, we were able to more deeply search for the possible influence of potentially moderating variables, such as sample ages or cognitive domain; second, our search was wide enough to almost completely avoid publication bias or other potentially threatening biases, except for parameter µ. On the other hand, one of the limitations of this meta-analysis is concurrent with its scope. Analysis of ex-Gaussian parameters, like any other approach using only RT distributions, do not take into account other indices, such as accuracy measures. Acknowledging the theoretical contributions of ex-Gaussian-based analyses and their usefulness in ADHD detection and more, approaches taking into account other performance variables are encouraged. Diffusion model are an instance of these approaches (Ging-Jehli et al., 2021; Huang-Pollock et al., 2017). Their use in various ambits helped disentangling the cognitive significance of the ex-Gaussian parameters (Matzke & Wagenmakers, 2009). Last, risk of bias assessment indicates a strong small-sample effect on µ estimation. This arises concerns on the reliability on findings based on parameter µ. Nonetheless, the potential bias direction has been taking into account, as it likely indicates that µ is lower for groups of people with ADHD than directly estimated, and this analysis was incorporated to our findings. Finally, despite having highlighted the advantages of using ex-Gaussian parameters, a limitation on the technical applicability of these findings must also be mentioned. To appropriately fitting ex-Gaussian distributions, a minimum of 100 trials per participant is necessary. Also, researches should quantitatively measure individual RT fits to ex-Gaussian models, because some RT distribution may exceptionally resemble Gaussian distribution, which compromises those particular model fits. Furthermore, sample distribution of σ and τ parameters, precisely the most important ones on ADHD discrimination, are not normal, and only with relatively large samples (i. e. >30 observers) these sample distributions approximate to Normal.

1. Conclusion

Estimating ex-Gaussian parameters for RT is a useful tool to understand the different cognitive profiles, and subsequently distinguish, between people with and without ADHD. τ and σ parameters are larger for groups with ADHD, and these parameters are predictably influenced by cognitive domain of tasks. Also, differences tend to be stable across ages. Parameter µ tends to be similar in both groups, but this similarity seems to be influenced by developmental stage and task.

1. References

Alderson, R. M., Kasper, L. J., Hudec, K. L., & Patros, C. H. (2013). Attention-deficit/hyperactivity disorder (ADHD) and working memory in adults: a meta-analytic review. *Neuropsychology*, *27*(3), 287.

Ali, S. (2020). *An investigation of the diagnostic utility of intraindividual variability in attention deficit/hyperactivity disorder: an ex-Gaussian approach*

Assink, M., & Wibbelink, C. J. (2016). Fitting three-level meta-analytic models in R: A step-by-step tutorial. *The Quantitative Methods for Psychology*, *12*(3), 154-174.

Barkley, R. A. (1997). Behavioral inhibition, sustained attention, and executive functions: constructing a unifying theory of ADHD. *Psychological bulletin*, *121*(1), 65.

Borella, E., De Ribaupierre, A., Cornoldi, C., & Chicherio, C. (2013). Beyond interference control impairment in ADHD: evidence from increased intraindividual variability in the color-stroop test. *Child Neuropsychology*, *19*(5), 495-515.

Brunkhorst-Kanaan, N., Verdenhalven, M., Kittel-Schneider, S., Vainieri, I., Reif, A., & Grimm, O. (2020). The Quantified Behavioral Test—A Confirmatory Test in the Diagnostic Process of Adult ADHD? *Frontiers in psychiatry*, *11*, 216.

Buzy, W. M., Medoff, D. R., & Schweitzer, J. B. (2009). Intra-individual variability among children with ADHD on a working memory task: an ex-Gaussian approach. *Child Neuropsychology*, *15*(5), 441-459.

Cai, W., Warren, S. L., Duberg, K., Pennington, B., Hinshaw, S. P., & Menon, V. (2021). Latent brain state dynamics distinguish behavioral variability, impaired decision-making, and inattention. *Molecular Psychiatry*, 1-14.

Castellanos, F. X., Sonuga-Barke, E. J., Milham, M. P., & Tannock, R. (2006). Characterizing cognition in ADHD: beyond executive dysfunction. *Trends in cognitive sciences*, *10*(3), 117-123.

Castellanos, F. X., Sonuga-Barke, E. J., Scheres, A., Di Martino, A., Hyde, C., & Walters, J. R. (2005). Varieties of attention-deficit/hyperactivity disorder-related intra-individual variability. *Biological psychiatry*, *57*(11), 1416-1423.

Cheung, M. W.-L. (2015). *Meta-analysis: A structural equation modeling approach*. John Wiley & Sons.

Cheung, M. W.-L., & Vijayakumar, R. (2016). A guide to conducting a meta-analysis. *Neuropsychology review*, *26*(2), 121-128.

Chiang, H.-L., Lin, H.-Y., Tseng, W.-Y. I., Hwang-Gu, S.-L., Shang, C.-Y., & Gau, S. S.-F. (2021). Neural substrates underpinning intra-individual variability in children with ADHD: A voxel-based morphometry study. *Journal of the Formosan Medical Association*.

Cohen, J. (1988). Statistical Power Analysis for the Behavioural Sciences (2nd ed.) Academic Press. *Orlando, Florida*.

Conn, V. S., Valentine, J. C., Cooper, H. M., & Rantz, M. J. (2003). Grey literature in meta-analyses. *Nursing research*, *52*(4), 256-261.

Dawson, M. R. (1988). Fitting the ex-Gaussian equation to reaction time distributions. *Behavior Research Methods, Instruments, & Computers*, *20*(1), 54-57.

Der, G., & Deary, I. J. (2003). IQ, reaction time and the differentiation hypothesis. *Intelligence*, *31*(5), 491-503.

Dotare, M., Bader, M., Mesrobian, S. K., Asai, Y., Villa, A. E., & Lintas, A. (2020). Attention Networks in ADHD Adults after Working Memory Training with a Dual n-Back Task. *Brain Sciences*, *10*(10), 715.

Duffy, K. A., Rosch, K. S., Nebel, M. B., Seymour, K. E., Lindquist, M. A., Pekar, J. J., . . . Cohen, J. R. (2021). Increased integration between default mode and task-relevant networks in children with ADHD is associated with impaired response control. *Developmental Cognitive Neuroscience*, 100980.

Epstein, J. N., Langberg, J. M., Rosen, P. J., Graham, A., Narad, M. E., Antonini, T. N., . . . Altaye, M. (2011). Evidence for higher reaction time variability for children with ADHD on a range of cognitive tasks including reward and event rate manipulations. *Neuropsychology*, *25*(4), 427.

Feige, B., Biscaldi, M., Saville, C. W., Kluckert, C., Bender, S., Ebner-Priemer, U., . . . Klein, C. (2013). On the temporal characteristics of performance variability in attention deficit hyperactivity disorder (ADHD). *PloS one*, *8*(10), e69674.

Fernández-Castilla, B., Declercq, L., Jamshidi, L., Beretvas, S. N., Onghena, P., & Van den Noortgate, W. (2021). Detecting selection bias in meta-analyses with multiple outcomes: a simulation study. *The Journal of Experimental Education*, *89*(1), 125-144.

Fitousi, D. (2020). Linking the ex-Gaussian parameters to cognitive stages: Insights from the linear ballistic accumulator (LBA) model. *Quant. Methods Psychol*, *16*, 91-106.

Galloway-Long, H., & Huang-Pollock, C. (2018). Using inspection time and ex-Gaussian parameters of reaction time to predict executive functions in children with ADHD. *Intelligence*, *69*, 186-194.

Galloway-Long, H., Huang-Pollock, C., & Neely, K. (2021). Ahead of the (ROC) Curve: A Statistical Approach to Utilizing Ex-Gaussian Parameters of Reaction Time in Diagnosing ADHD Across Three Developmental Periods. *Journal of the International Neuropsychological Society*, 1-14. <https://doi.org/10.1017/S1355617721000990>

Gaub, M., & Carlson, C. L. (1997). Gender differences in ADHD: A meta-analysis and critical review. *Journal of the American Academy of Child & Adolescent Psychiatry*, *36*(8), 1036-1045.

Gershon, J. (2002). A meta-analytic review of gender differences in ADHD. *Journal of attention disorders*, *5*(3), 143-154.

Geurts, H. M., Grasman, R. P., Verté, S., Oosterlaan, J., Roeyers, H., van Kammen, S. M., & Sergeant, J. A. (2008). Intra-individual variability in ADHD, autism spectrum disorders and Tourette's syndrome. *Neuropsychologia*, *46*(13), 3030-3041.

Ging-Jehli, N. R., Ratcliff, R., & Arnold, L. E. (2021). Improving neurocognitive testing using computational psychiatry—A systematic review for ADHD. *Psychological Bulletin*, *147*(2), 169.

Gmehlin, D., Fuermaier, A. B., Walther, S., Debelak, R., Rentrop, M., Westermann, C., . . . Tucha, O. (2014). Intraindividual variability in inhibitory function in adults with ADHD–an ex-Gaussian approach. *PloS one*, *9*(12), e112298.

Gmehlin, D., Fuermaier, A. B., Walther, S., Tucha, L., Koerts, J., Lange, K. W., . . . Aschenbrenner, S. (2016). Attentional lapses of adults with attention deficit hyperactivity disorder in tasks of sustained attention. *Archives of Clinical Neuropsychology*, *31*(4), 343-357.

Gooch, D., Snowling, M. J., & Hulme, C. (2012). Reaction time variability in children with ADHD symptoms and/or dyslexia. *Developmental Neuropsychology*, *37*(5), 453-472.

Halliday, D. W., Kim, Y., MacDonald, S. W., Garcia-Barrera, M. A., Hundza, S. R., & Macoun, S. J. (2021). Intraindividual variability in executive and motor control tasks in children with attention deficit hyperactivity disorder. *Journal of clinical and experimental neuropsychology*, *43*(6), 568-578.

Hasson, R., & Fine, J. G. (2012). Gender differences among children with ADHD on continuous performance tests: a meta-analytic review. *Journal of attention disorders*, *16*(3), 190-198.

Heathcote, A. (1996). RTSYS: A DOS application for the analysis of reaction time data. *Behavior Research Methods, Instruments, & Computers*, *28*(3), 427-445.

Heathcote, A., Popiel, S. J., & Mewhort, D. J. (1991). Analysis of response time distributions: an example using the Stroop task. *Psychological bulletin*, *109*(2), 340.

Henríquez-Henríquez, M. P., Billeke, P., Henríquez, H., Zamorano, F. J., Rothhammer, F., & Aboitiz, F. (2015). Intra-individual response variability assessed by ex-Gaussian analysis may be a new endophenotype for attention-deficit/hyperactivity disorder. *Frontiers in psychiatry*, *5*, 197.

Hernaiz-Guijarro, M., Castro-Palacio, J., Navarro-Pardo, E., Isidro, J., & Fernández-de-Córdoba, P. (2019). A probabilistic classification procedure based on response time analysis towards a quick pre-diagnosis of student’s attention deficit. *Mathematics*, *7*(5), 473.

Hervey, A. S., Epstein, J. N., Curry, J. F., Tonev, S., Eugene Arnold, L., Keith Conners, C., . . . Hechtman, L. (2006). Reaction time distribution analysis of neuropsychological performance in an ADHD sample. *Child Neuropsychology*, *12*(2), 125-140.

Huang-Pollock, C., Ratcliff, R., McKoon, G., Shapiro, Z., Weigard, A., & Galloway-Long, H. (2017). Using the diffusion model to explain cognitive deficits in attention deficit hyperactivity disorder. *Journal of abnormal child psychology*, *45*(1), 57-68.

Hwang, S. L. (2011). *Exploration of the Differences in Effort Regulation among ADHD subtypes - Based on ex-Gaussian Distribution* National ChengChi University]. Taipei, Taiwan.

Hwang-Gu, S.-L., Chen, Y.-C., Liang, S. H.-Y., Ni, H.-C., Lin, H.-Y., Lin, C.-F., & Gau, S. S.-F. (2019). Exploring the variability in reaction times of preschoolers at risk of attention-deficit/hyperactivity disorder: An ex-Gaussian analysis. *Journal of abnormal child psychology*, *47*(8), 1315-1326.

Hwang-Gu, S.-L., Gau, S. S.-F., Tzang, S.-W., & Hsu, W.-Y. (2013). The ex-Gaussian distribution of reaction times in adolescents with attention-deficit/hyperactivity disorder. *Research in developmental disabilities*, *34*(11), 3709-3719.

Hwang-Gu, S.-L., Lin, H.-Y., Chen, Y.-C., Tseng, Y.-h., Hsu, W.-Y., Chou, M.-C., . . . Gau, S. S.-F. (2019). Symptoms of ADHD Affect Intrasubject Variability in Youths with Autism Spectrum Disorder: An Ex-Gaussian Analysis. *Journal of Clinical Child & Adolescent Psychology*, *48*(3), 455-468.

Jacobson, L. A., Ryan, M., Denckla, M. B., Mostofsky, S. H., & Mahone, E. M. (2013). Performance lapses in children with attention-deficit/hyperactivity disorder contribute to poor reading fluency. *Archives of Clinical Neuropsychology*, *28*(7), 672-683.

Johnson, N. L., & Kotz, S. (1970). *Continuous Univariate Distributions* (Vol. 1). John Wiley & Sons.

Kaiser, S., Roth, A., Rentrop, M., Friederich, H.-C., Bender, S., & Weisbrod, M. (2008). Intra-individual reaction time variability in schizophrenia, depression and borderline personality disorder. *Brain and cognition*, *66*(1), 73-82.

Karalunas, S. L., Geurts, H. M., Konrad, K., Bender, S., & Nigg, J. T. (2014). Annual research review: Reaction time variability in ADHD and autism spectrum disorders: Measurement and mechanisms of a proposed trans‐diagnostic phenotype. *Journal of Child Psychology and Psychiatry*, *55*(6), 685-710.

Karalunas, S. L., & Huang-Pollock, C. L. (2013). Integrating impairments in reaction time and executive function using a diffusion model framework. *Journal of abnormal child psychology*, *41*(5), 837-850.

Karantinos, T., Tsoukas, E., Mantas, A., Kattoulas, E., Stefanis, N. C., Evdokimidis, I., & Smyrnis, N. (2014). Increased intra-subject reaction time variability in the volitional control of movement in schizophrenia. *Psychiatry research*, *215*(1), 26-32.

Kasper, L. J., Alderson, R. M., & Hudec, K. L. (2012). Moderators of working memory deficits in children with attention-deficit/hyperactivity disorder (ADHD): a meta-analytic review. *Clinical psychology review*, *32*(7), 605-617.

Keith, J. R., Blackwood, M. E., Mathew, R. T., & Lecci, L. B. (2017). Self-reported mindful attention and awareness, go/no-go response-time variability, and attention-deficit hyperactivity disorder. *Mindfulness*, *8*(3), 765.

Kofler, M. J., Rapport, M. D., Sarver, D. E., Raiker, J. S., Orban, S. A., Friedman, L. M., & Kolomeyer, E. G. (2013). Reaction time variability in ADHD: a meta-analytic review of 319 studies. *Clinical psychology review*, *33*(6), 795-811.

Kolodny, T., Mevorach, C., Stern, P., Ankaoua, M., Dankner, Y., Tsafrir, S., & Shalev, L. (2021). Are attention and cognitive control altered by fMRI scanner environment? Evidence from Go/No-go tasks in ADHD. *Brain imaging and behavior*, 1-11.

Kóbor, A., Takács, Á., Bryce, D., Szűcs, D., Honbolygó, F., Nagy, P., & Csépe, V. (2015). Children with ADHD show impairments in multiple stages of information processing in a Stroop task: An ERP study. *Developmental Neuropsychology*, *40*(6), 329-347.

Kölle, M., Mackert, S., Heckel, K., Philipsen, A., Ulrich, M., & Grön, G. (2022). Lower fractional anisotropy of the corticothalamic tract and increased response time variability in adult patients with ADHD. *Journal of Psychiatry and Neuroscience*, *47*(2), E99-E108.

Lacouture, Y., & Cousineau, D. (2008). How to use MATLAB to fit the ex-Gaussian and other probability functions to a distribution of response times. *Tutorials in quantitative methods for psychology*, *4*(1), 35-45.

Lee, R. W., Jacobson, L. A., Pritchard, A. E., Ryan, M. S., Yu, Q., Denckla, M. B., . . . Mahone, E. M. (2015). Jitter reduces response-time variability in ADHD: An ex-Gaussian analysis. *Journal of attention disorders*, *19*(9), 794-804.

Lenhard, W., & Lenhard, A. (2016). Psychometrica. Calculation of Effect Sizes. *Dettelbach (Germany).[accessed 2021 Oct 22].* [*https://www*](https://www)*. psychometrica. de/effect_size. html*.

Leth-Steensen, C., Elbaz, Z. K., & Douglas, V. I. (2000). Mean response times, variability, and skew in the responding of ADHD children: a response time distributional approach. *Acta psychologica*, *104*(2), 167-190.

Levy, F., Pipingas, A., Harris, E. V., Farrow, M., & Silberstein, R. B. (2018). Continuous performance task in ADHD: Is reaction time variability a key measure? *Neurpsychiatric Disease and Treatment*, *14*, 781-786.

Lin, H.-Y., Gau, S.-F., Huang-Gu, S., Shang, C.-Y., Wu, Y.-H., & Tseng, W.-Y. (2014). Neural substrates of behavioral variability in attention deficit hyperactivity disorder: based on ex-Gaussian reaction time distribution and diffusion spectrum imaging tractography. *Psychological Medicine*, *44*(8), 1751-1764.

Lin, H. Y., Hwang‐Gu, S. L., & Gau, S. F. (2015). Intra‐individual reaction time variability based on ex‐Gaussian distribution as a potential endophenotype for attention‐deficit/hyperactivity disorder. *Acta Psychiatrica Scandinavica*, *132*(1), 39-50.

Luce, R. D. (1986). *Response times: Their role in inferring elementary mental organization*. Oxford University Press on Demand.

Machida, K., Barry, E., Mulligan, A., Gill, M., Robertson, I. H., Lewis, F. C., . . . Johnson, K. A. (2022). Which Measures From a Sustained Attention Task Best Predict ADHD Group Membership? *Journal of Attention Disorders*, 10870547221081266.

Matthews, G., & Dorn, L. (1989). IQ and choice reaction time: An information processing analysis. *Intelligence*, *13*(4), 299-317.

Matzke, D., & Wagenmakers, E.-J. (2009). Psychological interpretation of the ex-Gaussian and shifted Wald parameters: A diffusion model analysis. *Psychonomic bulletin & review*, *16*(5), 798-817.

McAuley, L., Tugwell, P., & Moher, D. (2000). Does the inclusion of grey literature influence estimates of intervention effectiveness reported in meta-analyses? *The Lancet*, *356*(9237), 1228-1231.

Metin, B., Wiersema, J. R., Verguts, T., Gasthuys, R., van Der Meere, J. J., Roeyers, H., & Sonuga-Barke, E. (2016). Event rate and reaction time performance in ADHD: Testing predictions from the state regulation deficit hypothesis using an ex-Gaussian model. *Child Neuropsychology*, *22*(1), 99-109.

Morís Fernández, L., & Vadillo, M. A. (2020). Flexibility in reaction time analysis: many roads to a false positive? *Royal Society open science*, *7*, 190831.

Nakagawa, S., Lagisz, M., Jennions, M. D., Koricheva, J., Noble, D. W., Parker, T. H., . . . O'Dea, R. E. (2022). Methods for testing publication bias in ecological and evolutionary meta‐analyses. *Methods in Ecology and Evolution*, *13*(1), 4-21.

Nejati, V., & Yazdani, S. (2020). Time perception in children with attention deficit–hyperactivity disorder (ADHD): Does task matter? A meta-analysis study. *Child Neuropsychology*, *26*(7), 900-916.

Osmon, D. C., Kazakov, D., Santos, O., & Kassel, M. T. (2018). Non-Gaussian distributional analyses of reaction times (RT): improvements that increase efficacy of RT tasks for describing cognitive processes. *Neuropsychology review*, *28*(3), 359-376.

Passarelli, M., Piccinno, T. F., Ragazzo, F., Totino, A., & Benso, F. (2015). *Nuovi paradigmi statistici per la valutazione degli indici sensibili allo stato di ADHD* X Congresso Nazionale AIDAI-AIRIPA "Le Nuove Pratiche di Intervento per l'ADHD". Sarzana.

Patros, C. H., L. Sweeney, K., Mahone, E. M., Mostofsky, S. H., & Rosch, K. S. (2018). Greater delay discounting among girls, but not boys, with ADHD correlates with cognitive control. *Child Neuropsychology*, *24*(8), 1026-1046.

Polanczyk, G. V., Willcutt, E. G., Salum, G. A., Kieling, C., & Rohde, L. A. (2014). ADHD prevalence estimates across three decades: an updated systematic review and meta-regression analysis. *International journal of epidemiology*, *43*(2), 434-442.

Ptacek, R., Weissenberger, S., Braaten, E., Klicperova-Baker, M., Goetz, M., Raboch, J., . . . Stefano, G. B. (2019). Clinical implications of the perception of time in attention deficit hyperactivity disorder (ADHD): A review. *Medical science monitor: international medical journal of experimental and clinical research*, *25*, 3918.

Ratcliff, R., Schmiedek, F., & McKoon, G. (2008). A diffusion model explanation of the worst performance rule for reaction time and IQ. *Intelligence*, *36*(1), 10-17.

Rieger, T., & Miller, J. (2020). Are model parameters linked to processing stages? An empirical investigation for the ex-Gaussian, ex-Wald, and EZ diffusion models. *Psychological Research*, *84*(6), 1683-1699.

Rosch, K. S., Dirlikov, B., & Mostofsky, S. H. (2013). Increased intrasubject variability in boys with ADHD across tests of motor and cognitive control. *Journal of abnormal child psychology*, *41*(3), 485-495.

Rosenthal, R. (1995). Writing meta-analytic reviews. *Psychological bulletin*, *118*(2), 183.

Rousselet, G. A., & Wilcox, R. R. (2020). Reaction times and other skewed distributions: problems with the mean and the median. *Meta-Psychology*, *4*.

Ryan, M., Jacobson, L. A., Hague, C., Bellows, A., Denckla, M. B., & Mahone, E. M. (2017). Rapid automatized naming (RAN) in children with ADHD: An ex-Gaussian analysis. *Child Neuropsychology*, *23*(5), 571-587.

Ryan, M., Martin, R., Denckla, M. B., Mostofsky, S. H., & Mahone, E. M. (2010). Interstimulus jitter facilitates response control in children with ADHD. *Journal of the International Neuropsychological Society*, *16*(2), 388-393.

Salunkhe, G., Feige, B., Saville, C., Lancaster, T., Stefanou, M., Bender, S., . . . Linden, D. (2019). The impact of the COMT genotype and cognitive demands on facets of intra-subject variability. *Brain and cognition*, *132*, 72-79.

Salunkhe, G., Feige, B., Saville, C. W., Stefanou, M. E., Linden, D., Bender, S., . . . Klein, C. (2020). Dissociating slow responses from slow responding. *Frontiers in Psychiatry*, *11*, 943.

Salunkhe, G., Weissbrodt, K., Feige, B., Saville, C., Berger, A., Dundon, N., . . . Biscaldi, M. (2021). Examining the overlap between ADHD and autism spectrum disorder (ASD) using candidate endophenotypes of ADHD. *Journal of Attention Disorders*, *25*(2), 217-232.

Santerre-Lemmon, L. E. (2011). *Re-examining the Stop-Signal Task to test competing theories of AD/HD* University of Denver].

Sergeant, J. A. (2005). Modeling attention-deficit/hyperactivity disorder: a critical appraisal of the cognitive-energetic model. *Biological psychiatry*, *57*(11), 1248-1255.

Seymour, K. E., Mostofsky, S. H., & Rosch, K. S. (2016). Cognitive load differentially impacts response control in girls and boys with ADHD. *Journal of abnormal child psychology*, *44*(1), 141-154.

Shahar, N., Teodorescu, A. R., Karmon-Presser, A., Anholt, G. E., & Meiran, N. (2016). Memory for action rules and reaction time variability in attention-deficit/hyperactivity disorder. *Biological Psychiatry: Cognitive Neuroscience and Neuroimaging*, *1*(2), 132-140.

Siddaway, A. P., Wood, A. M., & Hedges, L. V. (2019). How to do a systematic review: a best practice guide for conducting and reporting narrative reviews, meta-analyses, and meta-syntheses. *Annual review of psychology*, *70*, 747-770.

Smyrnis, N., Karantinos, T., Malogiannis, I., Theleritis, C., Mantas, A., Stefanis, N. C., . . . Evdokimidis, I. (2009). Larger variability of saccadic reaction times in schizophrenia patients. *Psychiatry research*, *168*(2), 129-136.

Stasinopoulos, M., Rigby, B., Akantziliotou, C., Heller, G., Ospina, R., & Stasinopoulos, M. M. (2021). Package ‘gamlss. dist’. In.

Tamm, L., Narad, M. E., Antonini, T. N., O’Brien, K. M., Hawk, L. W., & Epstein, J. N. (2012). Reaction time variability in ADHD: a review. *Neurotherapeutics*, *9*(3), 500-508.

Tarantino, V., Cutini, S., Mogentale, C., & Bisiacchi, P. S. (2013). Time-on-task in children with ADHD: an ex-Gaussian analysis. *Journal of the International Neuropsychological Society*, *19*(7), 820-828.

Thissen, A. J., Luman, M., Hartman, C., Hoekstra, P., van Lieshout, M., Franke, B., . . . Buitelaar, J. K. (2014). Attention-deficit/hyperactivity disorder (ADHD) and motor timing in adolescents and their parents: familial characteristics of reaction time variability vary with age. *Journal of the American Academy of Child & Adolescent Psychiatry*, *53*(9), 1010-1019. e1014.

Tye, C., Johnson, K. A., Kelly, S. P., Asherson, P., Kuntsi, J., Ashwood, K. L., . . . McLoughlin, G. (2016). Response time variability under slow and fast‐incentive conditions in children with ASD, ADHD and ASD+ ADHD. *Journal of Child Psychology and Psychiatry*, *57*(12), 1414-1423.

Vainieri, I., Adamo, N., Michelini, G., Kitsune, V., Asherson, P., & Kuntsi, J. (2020). Attention regulation in women with ADHD and women with bipolar disorder: An ex-Gaussian approach. *Psychiatry research*, *285*, 112729.

van Belle, J., van Hulst, B. M., & Durston, S. (2015). Developmental differences in intra‐individual variability in children with ADHD and ASD. *Journal of Child Psychology and Psychiatry*, *56*(12), 1316-1326.

van Belle, J., van Raalten, T., Bos, D. J., Zandbelt, B. B., Oranje, B., & Durston, S. (2015). Capturing the dynamics of response variability in the brain in ADHD. *NeuroImage: Clinical*, *7*, 132-141.

Van den Noortgate, W., López-López, J. A., Marín-Martínez, F., & Sánchez-Meca, J. (2015). Meta-analysis of multiple outcomes: A multilevel approach. *Behavior research methods*, *47*(4), 1274-1294.

Van Zandt, T. (2000). How to fit a response time distribution. *Psychonomic bulletin & review*, *7*(3), 424-465.

Vaurio, R. G., Simmonds, D. J., & Mostofsky, S. H. (2009). Increased intra-individual reaction time variability in attention-deficit/hyperactivity disorder across response inhibition tasks with different cognitive demands. *Neuropsychologia*, *47*(12), 2389-2396.

Viechtbauer, W. (2010). Conducting meta-analyses in R with the metafor package. *Journal of statistical software*, *36*(3), 1-48.

Vinogradov, S., Poole, J. H., Willis-Shore, J., Ober, B. A., & Shenaut, G. K. (1998). Slower and more variable reaction times in schizophrenia: what do they signify? *Schizophrenia research*, *32*(3), 183-190.

Wagenmakers, E.-J., & Brown, S. (2007). On the linear relation between the mean and the standard deviation of a response time distribution. *Psychological review*, *114*(3), 830.

Wheaton, K. (2012). *Interference control and resource allocation in adults with attention-deficit/hyperactivity disorder: An evaluation of response variability and error compensation*. University of Rochester.

Wolfers, T., Onnink, A. M. H., Zwiers, M. P., Arias-Vasquez, A., Hoogman, M., Mostert, J. C., . . . Franke, B. (2015). Lower white matter microstructure in the superior longitudinal fasciculus is associated with increased response time variability in adults with attention-deficit/hyperactivity disorder. *Journal of psychiatry & neuroscience: JPN*, *40*(5), 344.

Yamashita, A., Rothlein, D., Kucyi, A., Valera, E. M., Germine, L., Wilmer, J., . . . Esterman, M. (2021). Variable rather than extreme slow reaction times distinguish brain states during sustained attention. *Scientific reports*, *11*(1), 1-13.

Zhao, Y., Nebel, M. B., Caffo, B. S., Mostofsky, S. H., & Rosch, K. S. (2021). Beyond massive univariate tests: Covariance regression reveals complex patterns of functional connectivity related to attention-deficit/hyperactivity disorder, age, sex, and response control. *Biological Psychiatry Global Open Science*.
