# Supplementary material for: Differences in Ex-Gaussian Parameters from Response Time Distributions Between Individuals with and Without Attention Deficit/Hyperactivity Disorder: A Meta-analysis
Source: Neuropsychol Rev. 2023 Mar 6;34(1):320–37. doi: 10.1007/s11065-023-09587-2 (PMC10920450; doi:10.1007/s11065-023-09587-2)
Supplement: Supplementary file 4 — Supplementary Material 4 [file 11065_2023_9587_MOESM4_ESM.docx]

Appendix 1: Sensitivity analysis

We found five outcomes from four different studies which reported a non-significant difference in µ between people with and without ADHD. We followed the recommendation from Rosenthal (1995) and assigned these studies an effect size of 0, rather than discarding them. It is possible that this decission alteres our findings. To check this possibility, we performed the same analysis removing these studies.

Figure S1 shows the forest and funnel plots of the effect size estimation without the studies with assigned sample size of 0. The estimated combined effect size is slightly larger than the main meta-analysis (0.0497 vs. 0.0447) and the assymetry in the funeel plot remains.


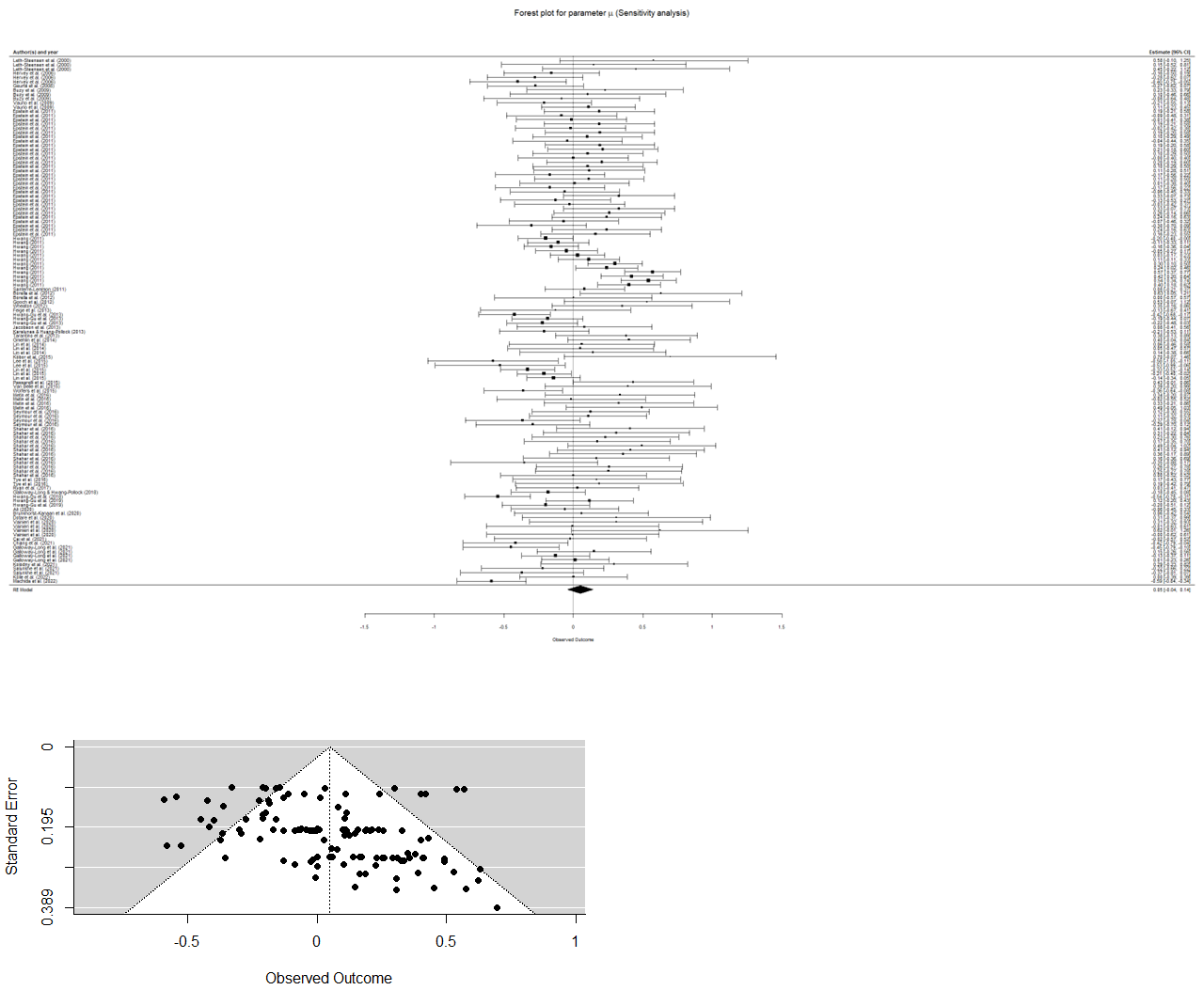


Figure S1: Forest and funnel plots for the effect size excluding studies reporting non-significant differences.

Similarly as the analysis with all the outcomes, the meta-regression with the square root of the effective set size is significant (QM(1) = 11.0772, p = 0.0009), and the overall estimate is also similar to the one obtained including those five outcomes (-0.1781 vs. -0.1724). Overall, setting those five outcomes to 0 has little impact on the results.
