# Supplementary material for: Differences in Ex-Gaussian Parameters from Response Time Distributions Between Individuals with and Without Attention Deficit/Hyperactivity Disorder: A Meta-analysis
Source: Neuropsychol Rev. 2023 Mar 6;34(1):320–37. doi: 10.1007/s11065-023-09587-2 (PMC10920450; doi:10.1007/s11065-023-09587-2)
Supplement: Supplementary file 5 — Supplementary Material 5 [file 11065_2023_9587_MOESM5_ESM.docx]

Appendix 2: Overlapping between samples

As exposed in the main text of this article, overlapping between two or more samples compromises the independence assumption

We considered that two studies had a potential sample overlapping when they shared at least one author or shared at least one author with another study already in a cluster. That way, we obtained seven clusters of studies with potential sample overlapping. Here they are described, together with the information collected and the decisions made, with no particular order.

Cluster 1: Harvey et al. (2006) and Epstein et al. (2011). The authors declared no overlapping between samples.

Cluster 2: Chiang et al. (2021), Hwang (2011), Hwang-Gu et al. (2013, 2018, 2019), Lin et al. (2014, 2015). No response from the authors. Overlapping assumed.

Cluster 3: Feige et al. (2013) and Salunkhe et al. (2021). The time lapse between studies is large, being the ages od the participants similar. Aldo, the researcher who collected data in Feige et al. (2013) is not involved in Salunkhe et al. (2021). Taken together, this evidence allows us to discard the possibility of overlapping or coincidence between samples.

Cluster 4: Duffy et al. (2021), Jacobson et al. (2013), Lee et al. (2015), Patros et al. (2018), Rosch et al. (2013), Ryan et al. (2017), Seymour et al. (2016), Vaurio et al. (2009), Zhao et al. (2021). No responde from the autors. Overlapping assumed.

Cluster 5: Gmehlin et al. (2014) and Gmehlin et al. (2016). The authors declared the studies used different samples at different time points.

Cluster 6: Brunkhorst-Kanaan et al. (2020), Tye et al. (2016), and Vainieri et al. (2020). The authors declared no overlapping between samples.

Cluster 7: Galloway-Long & Huang-Pollock (2018), Galloway-Long et al. (2021), Karalunas & Huang-Pollock (2013). The authors reported two-way overlapping proportions between 15% and 35%.

We decided to treat clusters 2, 4, and 7 as study clusters and clusters 1, 3, 5, and 6 as independent studies.
